# AI-enabled eye-movement and emerging multimodal frameworks for precision dyslexia screening and reading pattern analysis

**DOI:** 10.3389/fmed.2026.1847464

**Published:** 2026-06-19

**Authors:** Ashit Kumar Dutta, Moattar Raza Rizvi, Farha Mujeeb Ahmed Shaikh, Adel Mefleh Widyan

**Affiliations:** 1 Department of Computer Science and Information Systems, College of Medical Sciences, AlMaarefa University, Dariyah, Saudi Arabia; 2 Faculty of Allied Health Sciences, Santosh Deemed to be University, Ghaziabad, India; 3 Department of Basic Medical Science, College of Medicine, AlMaarefa University, Diriyah, Riyadh, Saudi Arabia; 4 King Salman Center for Disability Research, Riyadh, Saudi Arabia; 5 Department of Mathematics, College of Science, Qassim University, Buraydah, Saudi Arabia

**Keywords:** automated screening, dyslexia, electrooculography, eye movement, eye tracking, machine learning, reading behavior

## Abstract

**Introduction:**

Developmental dyslexia refers to a common neurodevelopmental disorder, which impairs the accuracy and fluency of reading, and early identification is vital for initiating timely intervention. Nonetheless, the traditional methods of formal assessment are time- and resource-intensive, which limits their scalability. Machine-learning approaches and eye-tracking technologies provide objective, data-driven solutions for dyslexia screening. This research integrates current evidence on eye-movement-based and emerging multimodal computational methods for dyslexia screening, risk identification, and algorithmic classification during reading tasks.

**Methods:**

PubMed, Scopus, Web of Science, and CINAHL were searched systematically to identify studies published between January 2015 and March 2026. Eligible studies included analysis of eye-movement obtained via eye tracking or electrooculography (EOG), with or without predictive modeling. Methodological quality was assessed using JBI, PROBAST, ROBINS-I, and COSMIN tools.

**Results:**

Twenty-three articles were included out of 50 full-text articles screened comprising eye-movement biomarker/observational studies (*n* = 5), machine-learning prediction-model studies (*n* = 14), intervention response studies (*n* = 2), and reliability/feasibility studies (*n* = 2). The sample sizes ranged from small experimental cohorts (<20 participants) to larger datasets (>300 participants). In the literature, dyslexic readers were consistently found to exhibit longer fixation durations, increased regression behavior and reduced saccadic efficiency. Machine-learning algorithms using fixation, saccade, scan path, and signal-based features demonstrated classification accuracies ranging from approximately 80 to 95% with some studies reporting values approaching 99% under specific experimental conditions.

**Discussion:**

Nevertheless, there was a significant heterogeneity in datasets, feature extraction methods, outcome definitions and validation schemes. Notably, numerous studies used proxy diagnostic labels, small or internally derived datasets, and internal cross-validation, which introduces the risk of overfitting and performance inflation. Explicit multimodal or multi-source modeling was identified in three of 23 studies involving combinations of gaze data with demographic, cognitive, linguistic, VR-bed, text-derived, saliency-map, or CNN-based features. Two additional studies used EOG as an alternative eye-movement signal modality rather than true multi-source fusion. Therefore, the evidence base remains dominated by eye-movement and gaze-derived approaches, while multimodal evidence should be interpreted as emerging and exploratory. Altogether, eye-movement based computational systems are a promising, non-invasive method for scalable dyslexia screening.

**Systematic review registration:**

PROSPERO, identifier (RD42061332527).

## 1 Introduction

Developmental dyslexia is one of the most common neurodevelopmental disorders affecting reading acquisition and literacy development (1). It is characterized by persistent difficulties in accurate and fluent word recognition, spelling, and decoding that cannot be explained by general intelligence, inadequate educational opportunities, or sensory impairments (2). Dyslexia occurs across languages and orthographic systems and is widely recognized as a prevalent learning disability among school-aged children (3). Epidemiological estimates indicate that dyslexia affects a substantial proportion of the population, posing significant challenges for educational systems and healthcare professionals (4, 5). Early identification is critical, as delays in reading development are associated with long-term academic difficulties, reduced self-confidence, and an increased risk of emotional and behavioral problems later in life (6). However, early recognition in practice is often constrained by limited access to specialist assessment, variability in teacher training, and the time and personnel demands of comprehensive diagnostic pathways, particularly in large educational systems (7). From a paediatric screening perspective, gaze-based and computational approaches should be considered adjunctive risk-identification tools rather than replacements for comprehensive clinical or educational assessment, because screening aims to identify children who may require further evaluation, whereas formal confirmation of dyslexia requires standardized assessment and professional interpretation (8–10).

Reading is a complex cognitive process requiring coordinated interaction among visual perception, attention, language processing, and working memory (11). During reading, the eyes move across text through rapid saccades interspersed with fixations, during which visual and linguistic information is processed. Eye-movement behavior during reading reflects real-time cognitive processing demands, including lexical access, syntactic integration, and higher-level comprehension processes (12–14). Because gaze behavior can be continuously recorded while a reader interacts with text, eye-movement measures provide objective indicators of reading processes and may reveal processing inefficiencies not fully captured by conventional measures such as reading accuracy or speed (15).

Compared with skilled readers, individuals with dyslexia are frequently reported to exhibit longer fixation durations and increased regression behavior during reading (16). They may also demonstrate less efficient saccadic progression and disrupted visual scanning patterns while processing text (17). These gaze patterns are generally interpreted as reflecting increased processing demands during decoding and word recognition, although their magnitude may vary depending on language characteristics and experimental paradigms (18). Importantly, these gaze features should not be interpreted as causal factors of dyslexia but rather as observable correlates of underlying cognitive and linguistic constraints influencing real-time reading processes.

Eye-tracking technologies enable high-resolution measurement of gaze behavior and provide quantitative parameters such as fixation duration, fixation count, saccadic amplitude, and regression-related metrics that reflect reading-related visual and cognitive processing. In dyslexia research, these measures have been used to characterize atypical reading patterns and support risk identification during reading tasks (19–22). Advances in portable and tablet-based gaze-estimation systems may further expand feasibility in educational and clinical screening contexts, although reliability, calibration stability, and validity must be established before routine implementation (22, 23). These developments also introduce important methodological considerations that can influence data quality and interpretability, including sampling frequency, calibration stability, tolerance to head movement, stimulus presentation formats (e.g., screen versus paper, scrolling versus paginated text), and standardized procedures for handling tracking loss and exclusions.

In parallel, machine-learning and deep-learning methods have created new opportunities for analysing complex gaze-derived reading datasets. In dyslexia-specific research, computational models have used fixation, saccade, regression, scanpath, and interest-area features to support dyslexia-risk prediction, screening, or algorithmic classification using approaches such as support vector machines, random forests, multilayer perceptrons, convolutional neural networks, and interpretable eye-tracking feature models (24–28). These studies suggest that gaze-derived features can support automated risk classification; however, reported model performance is often sensitive to methodological factors, including the definition of outcome labels, feature engineering pipelines, participant-level data splitting, and the rigor of validation procedures. These concerns are consistent with prediction-model reporting and appraisal guidance, which emphasizes transparent model development, appropriate validation, and careful assessment of applicability before clinical or educational translation (29, 30). These issues are particularly relevant in eye-tracking datasets, which often include repeated observations per participant and may lead to inflated performance estimates if training and testing data are not appropriately separated.

More recently, a smaller body of dyslexia-focused work has examined multi-source approaches that combine gaze measures with demographic, cognitive, linguistic, behavioral, or physiological indicators. In the present review, explicit multimodal or multi-source modeling was identified in 3 of 23 studies. Shalileh et al. combined eye-movement data with demographic and non-verbal intelligence variables, Pereira et al. integrated eye-tracking with cognitive and linguistic predictors, and Vaitheeshwari et al. explored a VR-based fusion model combining eye-movement metrics with text-derived and saliency-map representations (31–33). These approaches may capture complementary information across multiple data sources, but they also increase system complexity, cost, missing-data risk, and validation requirements. Therefore, balancing predictive performance with feasibility remains a key consideration in the development of multimodal dyslexia screening systems.

From a precision-medicine perspective, gaze-based and multimodal computational approaches may contribute to more individualized pathways for identifying and supporting children with reading difficulties. Rather than treating dyslexia as a uniform condition, eye-movement features may help characterize individual differences in reading behavior, such as delayed fixation, excessive rereading, inefficient saccadic progression, reduced reading speed, or atypical scanpath organization. When combined with reading-performance measures, cognitive indicators, speech or oral-reading features, and physiological signals such as EOG, these data may support personalized risk profiling and phenotype stratification. Such stratification could help distinguish children whose reading difficulties are dominated by decoding inefficiency, visual-attentional instability, slow reading fluency, oculomotor control differences, or mixed profiles. In this way, computational analysis of reading behavior may contribute not only to screening or classification, but also to precision educational healthcare pathways in which risk identification, referral, intervention planning, and follow-up monitoring are tailored to the individual learner.

Despite the growing body of research, the literature remains fragmented across disciplines, including cognitive science, neuroscience, computer science, and educational technology. Substantial variability exists in participant characteristics, orthographic systems, reading paradigms, eye-tracking devices, feature extraction strategies, and machine-learning validation practices across dyslexia-focused eye-movement studies (19–21, 31). Differences in orthographic transparency across languages can also influence reading acquisition, eye-movement patterns and the relevance of specific gaze features for dyslexia screening (34, 35). Furthermore, many prediction-model studies rely on screening-based proxy labels rather than clinically confirmed diagnoses, and external validation remains limited. These methodological differences complicate direct comparison across studies and may affect the generalizability of proposed screening approaches. Consequently, a systematic synthesis is required to clarify methodological trends, identify commonly reported eye-movement indicators, and evaluate computational approaches for dyslexia screening, risk identification, and algorithmic classification.

Given these considerations, the aim of this systematic review is to synthesize current evidence on eye-movement measures and computational approaches used for dyslexia screening, risk identification, and algorithmic classification during reading-related tasks. Specifically, the review examines (i) commonly reported eye-movement features, (ii) eye-tracking and EOG technologies and experimental paradigms, (iii) machine-learning and deep-learning methods and their validation strategies, and (iv) emerging multimodal approaches that integrate gaze data with additional behavioral or physiological signals. By integrating evidence across these domains, this review aims to clarify current methodological practices and highlight priorities for the development of reliable and scalable dyslexia screening frameworks.

## 2 Materials and methods

### 2.1 Study design

This work was conducted as systematic review synthesizing evidence on dyslexia screening, risk identification, and algorithmic classification based on eye-movement and reading-behavior analysis, including computational approaches applied to gaze-derived data. The review was conducted and reported in accordance with PRISMA recommendations to support transparent reporting of the identification, screening, eligibility assessment, and synthesis processes (36). The protocol for this systematic review was prospectively registered in the International Prospective Register of Systematic Reviews (PROSPERO; Registration ID: CRD420261332527), and the review was conducted in accordance with the registered protocol. General methodological principles for systematic evidence synthesis were additionally informed by guidance from the Cochrane Handbook (37).

### 2.2 Search strategy

A comprehensive search strategy was developed to identify studies evaluating dyslexia screening, risk identification, or algorithmic classification using eye-movement analysis and reading-behavior monitoring, including computational approaches applied to gaze-derived data. Electronic searches were conducted in PubMed, Scopus, Web of Science, and CINAHL to capture literature spanning cognitive science, educational research, health sciences, and computer science. The search included studies published between January 2015 and March 2026, and the final database search was conducted in March 2026.

The search strategy combined controlled vocabulary terms (e.g., MeSH terms in PubMed) with free-text keywords related to dyslexia or reading difficulty, eye-movement, and gaze-based measurement, electrooculography, machine learning, artificial intelligence, classification, prediction, screening, and related database terms for detection and diagnosis. Boolean operators were used to combine terms, and the syntax was adapted for each database. The complete database-specific search strategies, including full Boolean search strings, search dates, applied limits, and records from PubMed/MEDLINE, Scopus, Web of Science, and CINAHL, are provided in Supplementary file 1. The database searches identified 1860 records in total before exclusions: Scopus (*n* = 1,215), PubMed/MEDLINE (*n* = 104), CINAHL (*n* = 105), and Web of Science (*n* = 436). The search was restricted to peer-reviewed articles published in English due to feasibility constraints related to translation resources. Grey literature was not searched because the review was restricted to peer-reviewed journal articles. In addition, the reference lists of relevant review articles and included studies were screened to identify potentially eligible articles not captured through database searching.

### 2.3 Eligibility criteria

Eligibility criteria were determined before the screening process began and were applied consistently throughout study selection. Studies were considered eligible if they investigated dyslexia screening, or risk identification, or algorithmic classification using eye-movement data collected during reading activities. The population of interest included children, adolescents, or adults diagnosed with developmental dyslexia or assessed for reading difficulties using standardized evaluation methods. Eligible studies were required to analyze eye-movement behavior recorded through eye-tracking devices, electrooculography (EOG), or comparable gaze-monitoring technologies while participants performed reading tasks. When available, studies that included comparisons between individuals with dyslexia and typically developing readers were included to enable examination of group differences in reading behavior.

Studies were also eligible if they reported outcomes related to dyslexia-risk identification, algorithmic classification, or characterization of dyslexia-related reading behavior based on eye-movement features, including approaches that used machine-learning, artificial intelligence, or statistical modeling techniques. Empirical study designs such as observational investigations, controlled experiments, prediction-model development studies, and intervention studies reporting eye-movement outcomes were included.

Studies were excluded if they did not examine eye-movement behavior during reading tasks, focused exclusively on treatment interventions without analyzing reading-behavior data, or presented theoretical or conceptual discussions without empirical evidence. Editorials, commentaries, dissertations, non-peer-reviewed reports, and conference abstracts without full articles were also excluded. In addition, studies examining visual impairments unrelated to dyslexia or studies that did not evaluate reading behavior during text processing were not considered eligible.

For terminology consistency, “screening” was used to describe approaches intended to identify individuals who may be at risk of dyslexia and require further assessment. “Classification” was used to describe algorithmic grouping or model-based prediction of dyslexia-related status. “Diagnosis” was reserved for formal clinical confirmation based on standardized assessment procedures or professional evaluation. The broader term “detection” was used only when referring to terminology used by original studies or database search terms; model outputs were not interpreted as clinical diagnoses unless dyslexia status was formally confirmed in the original study.

### 2.4 Study selection process

**Figure 1:**
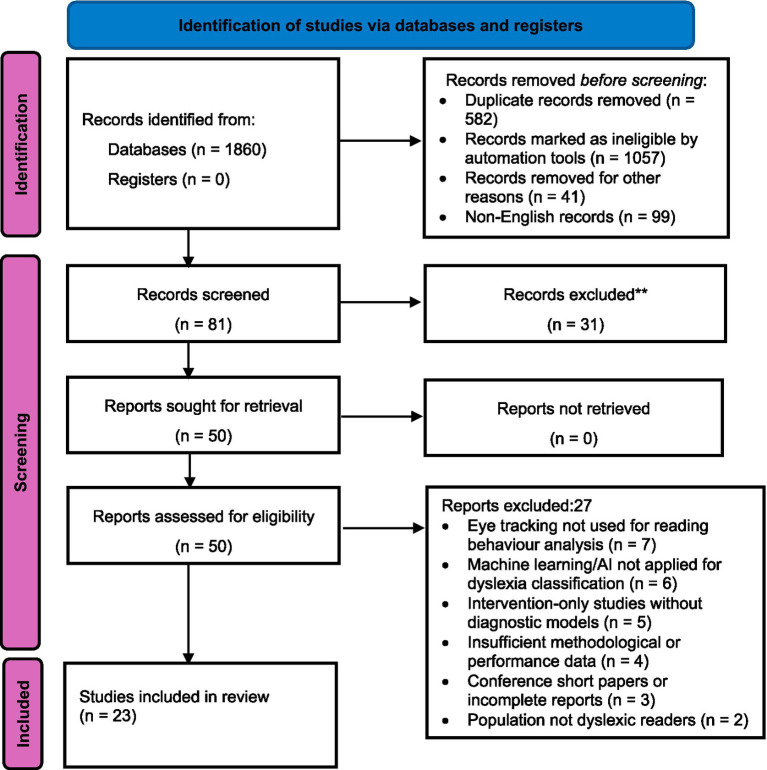
PRISMA 2020 flow diagram illustrating the study selection process for studies examining dyslexia detection and reading behavior using eye-tracking, electrooculography, and computational approaches during reading tasks.

Study identification and screening were conducted following the PRISMA 2020 framework. Title/abstract screening and full-text eligibility assessment were conducted independently by two reviewers, with disagreements resolved through discussion or consultation with a third reviewer. Records retrieved from PubMed, Scopus, Web of Science, and CINAHL were exported and duplicates were removed prior to the screening stage. Titles and abstracts were first reviewed to exclude clearly irrelevant reports, such as studies involving non-dyslexia populations, non-reading tasks, or non-empirical publications. Articles that appeared potentially eligible were then retrieved in full text and evaluated against the predefined inclusion and exclusion criteria. After screening, 50 full-text articles were assessed for eligibility, of which 23 studies met the inclusion criteria and were included in the qualitative synthesis. The complete selection procedure is illustrated in the PRISMA flow diagram (Figure 1).

### 2.5 Data extraction

Data extraction was conducted using a standardized data collection form to ensure consistent capture of relevant information across studies. The form was developed prior to extraction and applied uniformly during the review process. Two reviewers independently extracted data from each included study, and any discrepancies were resolved through discussion, with consultation from a third reviewer when necessary. Extracted information included bibliographic details (author, year, and country), study design, participant characteristics, sample size, and group definitions. Additional methodological and technical variables were recorded, including eye-tracking or sensor modality, reading paradigm, stimulus type, and reported eye-movement measures such as fixation duration and count, saccadic amplitude, regression-related indices, gaze or trajectory metrics, and reading speed where applicable.

For studies developing prediction models, information on model type, feature sets, validation approaches, and reported performance metrics was also extracted. Reported performance outcomes included measures such as accuracy, sensitivity, specificity, and area under the receiver operating characteristic (ROC) curve. Model validation strategies were systematically extracted and categorized as (i) internal validation (e.g., k-fold cross-validation or random train–test splits), (ii) participant-wise validation (ensuring separation of data at the subject level), and (iii) external validation using independent datasets. This classification was used to assess the robustness and generalizability of reported model performance.

In addition, studies were categorized based on the nature of dyslexia labels as either clinically validated or proxy labels. Clinically validated labels were defined as those derived from formal diagnostic procedures, including standardized clinical assessments or diagnosis by qualified professionals. Proxy labels referred to classifications based on screening thresholds, performance-based groupings, or dataset-derived labels without explicit clinical confirmation. This distinction was used during synthesis to interpret variability in model performance and generalizability.

### 2.6 Quality assessment

The methodological quality and risk of bias of included studies were assessed using appraisal tools appropriate to the respective study designs. Observational eye-tracking studies were evaluated using the Joanna Briggs Institute (JBI) critical appraisal checklist, which is commonly applied for assessing methodological rigor in observational research (38). Studies developing or evaluating prediction models were assessed using the Prediction model Risk of Bias Assessment Tool (PROBAST), which evaluates potential bias across four domains: participants, predictors, outcomes, and statistical analysis (39). Intervention or training studies were assessed using the ROBINS-I tool, which is designed for evaluating risk of bias in non-randomized intervention studies (40). Where randomized designs were identified, appropriate risk-of-bias considerations consistent with randomized study appraisal frameworks were applied. Quality assessment was performed independently by two reviewers, and any disagreements were resolved through discussion, with consultation from a third reviewer when necessary. The results of the risk-of-bias assessments were summarized in both tabular and narrative formats.

### 2.7 Data synthesis

Given substantial variation across studies in participant characteristics, languages and orthographies, reading paradigms (e.g., silent versus aloud reading; sentence versus passage reading; task constraints), device modalities and sampling rates, feature definitions, and validation approaches, a quantitative meta-analysis was not considered appropriate. Additional variability in machine-learning methodologies, dataset characteristics, and model validation approaches further limited the comparability of reported performance metrics across studies. Consequently, findings were synthesized using a structured narrative approach.

For synthesis, studies were grouped according to study objective and evidence type into four categories: (i) eye-movement biomarker/observational studies, including observational and experimental studies characterizing dyslexia-related reading behavior; (ii) machine-learning and artificial-intelligence prediction-model studies evaluating dyslexia-risk prediction, screening, or algorithmic classification performance; (iii) intervention response studies examining changes in reading performance or eye-movement outcomes following training or therapeutic interventions; and (iv) reliability and feasibility studies evaluating the stability, usability, or practical implementation of gaze-based screening approaches. Across these groups, we summarized commonly reported eye-movement features, eye-tracking devices and experimental paradigms, computational models, validation strategies, and reliability or feasibility findings. Where applicable, methodological strengths and limitations across studies were also compared to support interpretation of findings. Study selection counts are presented in the PRISMA flow diagram (Figure 1), and key extracted study characteristics are summarized in Tables 1–5.

**Table 1:** Characteristics of included studies by evidence type.

S. no.	Study (author, year, country)	Study design	Sample size and participants	Technology/method	Reading task	Eye-movement features	Key findings
Eye-movement biomarker/observational studies							
1	Gran Ekstrand et al., 2021, Sweden	Observational case-study (43)	*n* = 8; children 9-10y (at-risk by screener)	Tobii screen-based ET (120 Hz) + screening framework	Whole-passage reading (school setting)	Fixation duration; saccade length; regressions	Supports ET screening: at-risk children had poorer reading/decoding; some co-occurring attention issues
2	Rossier-Bisaillon et al., 2025, Canada	Observational case–control (42)	24 adults (10 DD, 14 controls), 18-56y	EyeLink 1,000 Plus (1,000 Hz) + eye-voice alignment	Standardized French text reading aloud (Alouette-R)	Word fixation probability; first-pass fixations; regressive fixation probability; FFD; gaze duration; eye-voice span	Dyslexia showed slower reading, more errors, atypical fixation patterns and reduced/unstable eye-voice span
3	Scaltritti et al., 2019, Italy	Observational Study (webpage reading; LME modeling) (44)	*N* = 79 total (adult/child × DD/control groups)	EyeLink 1,000 Plus (1,000 Hz)	Silent reading of real webpages + comprehension questions	Avg fixation duration; number of fixations; avg. saccade amplitude	Typography (font size, alignment, headers etc.) influenced reading; DD groups showed longer/more fixations and smaller saccades
4	Holmqvist Olander et al., 2017, Sweden	Experimental within-subject eye-tracking comparison (text-only vs. text+picture) (49)	*N* = 46 (19 DD, 27 controls) young adults	SMI RED250 (250 Hz)	Expository texts with/without illustration + questions	Dwell time; fixation duration/count; TTFF to picture; transition rate (text↔picture); scanpaths	Pictures did not improve comprehension for DD; DD tended to delay/neglect picture and switch less efficiently
5	Rossier-Bisaillon et al., 2026, Canada	Experimental eye-tracking study using gaze-contingent moving-window paradigm (41)	38 children (native French speakers), 9–12 years: 14 dyslexic + 24 age-matched controls	Gaze-contingent eye tracking (moving-window technique)	Children read sentences aloud while the number of visible characters around fixation was manipulated across 5 conditions: baseline (no restriction), 10-, 7-, 5-, and 3-character windows	Fixation duration, saccade length (plus reading speed & accuracy outcomes)	Window-size reduction had a diminished effect on reading speed in dyslexia vs. controls; eye data support increased foveal load and reduced parafoveal processing in dyslexic children; reading accuracy not affected by window size
Machine learning/Ai prediction-model studies for dyslexia detection							
6	Raatikainen et al., 2021, Finland	Prediction-model development study (20)	*N* = 161 students (~12.5y); labels via reading fluency cutoff	EyeLink 1,000 (1,000 Hz); RF feature selection + SVM	Information-search “question page” reading (10 trials)	Fixation duration/count; saccade amplitude/duration; AOI transition matrices; histogram features	Best hybrid RF → SVM reached ~89.7% accuracy; recall ~84.8% for dysfluent readers
7	Appadurai and Bhargavi, 2021, India	Machine-learning classification study (27)	185 children (9-10y; Benfatto dataset)	Ober-2 ET; I-DA/I-VA events; SVM-PSO, XGBoost, CNN (images)	Passage reading + comprehension	Fixation/saccade event features; scanpath length; (image-based fixation/scanpath)	Hybrid kernel SVM-PSO reported top accuracy (~96%); XGBoost ~95%
8	Nilsson Benfatto et al., 2016, Sweden	Machine-learning classification study (19)	185 children (97 high-risk, 88 low-risk)	Ober-2 (100 Hz); linear SVM + SVM-RFE	Silent short passage + comprehension	168 low-level fixation/saccade parameters (progressive/regressive; horiz/vert; version/vergence)	Best accuracy ~95.6% (balanced sensitivity/specificity)
9	Nerušil et al., 2021, Slovakia	Deep-learning classification study (26)	185 children (Benfatto dataset)	CNNs on horizontal gaze time-series/spectral magnitude	Text reading task (shared dataset)	Raw x-coordinate signal; magnitude spectrum (DFT)	Best CNN on spectral features ~96.6% accuracy
10	Pereira et al., 2024, Portugal	Predictive modeling study (32)	*N* = 59 children (Control, DD, ADHD-I groups)	Eye tracking + cognitive predictors	Silent reading with word length/frequency manipulation	Fixation count; SFD; FPRT; SPRT; TFT	Model reported ~81% correct classification; DD/ADHD-I linked to distinct cognitive + FC predictors
11	Shalileh et al., 2023, Russia	Machine-learning classification study (31)	*N* = 307 pupils (TD/DR/DD)	EyeLink 1,000 Plus/Portable Duo (1,000 Hz) + ML (MLP etc.)	Silent reading of 30 sentences (+ comprehension subset)	Fixation duration, coordinates; word-level IA metrics (FFD, TRT, FC, regressions, skip, etc.)	Best MLP models reported very high F1/ROC-AUC (≈0.91–0.93/≈0.98–0.99)
12	Vaitheeshwari et al., 2024, Taiwan	Pilot multimodal AI study (33)	10 DD + 4 controls	HTC Vive Pro + Tobii VR4 (120 Hz); ML + BERT + CNN (maps) + fusion	VR reading (multi-page; comprehension)	Fixation duration/count; saccade amplitude; trajectories; saliency maps	Fusion approach reported very high accuracy on pilot dataset (~98%)
13	Rello and Ballesteros, 2015, USA/Spain	Machine-learning classification study (54)	*N* = 97 (48 DD, 49 control), ages 11–54	Tobii 1750; polynomial SVM	12 short texts × 12 typefaces (+ comprehension control)	Reading time (sum of visits); mean fixation; # fixations (+ age)	Best accuracy ~80.18% (10-fold CV with subject-wise separation)
14	İleri et al., 2025, Türkiye	EOG-based deep-learning study (46)	23 DD + 13 controls (8–10y)	BIOPAC MP-36 EOG (100 Hz); CWT scalograms; CNN (DyslexiaNet etc.)	Read-aloud Turkish texts varied by typeface/font size	Reading time; blink rate; regression rate; EOG energy; scalogram inputs	DyslexiaNet reported very high accuracy (esp. horizontal channel); typeface effects reported
15	Bhargavi and Jothi Prabha, 2020, India	Machine-learning classification study (25)	Benfatto dataset (paper reports 187 but groups align with 185)	Ober-2 signals; I-DT/I-VT; Hybrid SVM-PSO; external validation mentioned	Reading task (inherited dataset)	Fixation duration/count; gaze duration proxy; saccade amplitude/duration; scanpath metrics; blinks	Hybrid SVM-PSO reported ~95.6% accuracy; external “real-time” validation reported ~96%
16	Latifoglu et al., 2021, Türkiye	Deep-learning signal classification study (47)	10 DD + 10 controls (8–12y)	BIOPAC MP36 EOG (100 Hz); STFT spectrograms + 2D-CNN	Reading 5-line text; events: re-reading vs. line skipping	EOG-derived rereading/line-skipping event segments	2D-CNN classified rereading vs. line-skipping with ~99% accuracy (movement-type recognition)
17	Vajs et al., 2022, Serbia	Machine-learning feature-engineering study (21)	30 children (15 DD, 15 control); 378 trials	SMI RED-m (120 Hz)	Silent reading under 13 color configurations	Fixation fractal dimension; fixation intersection coefficient/variability; active reading time; saccade variability + conventional metrics	Best ACC ~ 0.94 (LR) using proposed gaze-complexity features
18	Vajs et al., 2023, Serbia	Interpretable machine-learning study (28)	Same 30-child dataset; 13 color configs	SMI RED-m (60 Hz; also 30 Hz simulation); no fixation parsing	Silent reading	Self-intersection (SI) trigger feature; Vertical Alteration Score (VAS) feature	Best ACC ~ 88.9% (60 Hz) and ~87.8% (30 Hz) using single-feature models
19	Svaricek et al., 2025, Czech Republic	Deep-learning classification study (51)	35 pupils (13 DD, 22 controls)	SMI RED 250 (250 Hz); Fix-images + ResNet18 + ensemble	3 tasks (at-level, below-level, pseudo-text), read aloud 2 min each	Fixation duration + dispersion X/Y + position (Fix-image); baseline conventional metrics	Ensemble accuracy ~86.65%; cross-dataset test ~86.11%
Reliability and feasibility studies							
20	Le et al., 2023, Vietnam	Pilot observational system-evaluation study (50)	*N* = 15 (3 DD, 12 typical), ~2nd grade	Tobii 4C (90 Hz) + OCR-based AOIs	Vietnamese pseudowords/words + paragraph + comprehension	Fixation duration/count (AOI); scanpath/heatmaps	DD showed irregular scanpaths and dispersed/overlapping fixations vs. typical readers
21	Park et al., 2024, Korea	Test–retest reliability study (22)	*N* = 200 children, 8-13y	Tablet app + VisualCamp SeeSo (front-camera gaze estimation)	Grade-appropriate passages (test–retest)	Reading speed by gaze; mean fixation time; fixation frequency; saccadic length; regression ratio	Good-excellent reliability for fixation metrics and gaze-based reading speed
Intervention Response Studies							
22	Virlet et al., 2024, France	Pre–post intervention study (45)	Dyslexia *N* = 19 (ST *n* = 9; PSI *n* = 10) + controls *n* = 9	EyeLink 1,000 + PSI package (prism/oral stimulation/insoles/breathing)	Silent story reading + comprehension	FFD; gaze duration; saccade amplitude	PSI group showed larger gains in reading and “more typical” eye-movement changes vs. ST
23	Peters et al., 2021, Australia	Randomized controlled trial (48)	*N* = 64 dyslexic children (8-13y)	Fruit Ninja training; Gazepoint GP3HD (150 Hz) in AVG+	Reading outcomes (YARC) + RAN eye-tracking	Fixation duration; fixation count; regression count (during RAN)	Both AVG groups improved reading; eye-control did not add benefit over standard AVG

ADHD-I, attention-deficit/hyperactivity disorder—predominantly inattentive presentation; AI, artificial intelligence; AOI, area of interest; AVG, action video game; AVG-R, action video game training with standard mouse control; AVG+, action video game training with eye-gaze cursor control; BERT, Bidirectional Encoder Representations from Transformers; CNN, convolutional neural network; CV, cross-validation; CWT, continuous wavelet transform; DD, developmental dyslexia; DFT, discrete Fourier transform; EOG, electrooculography; ET, eye tracking; F1, F1-score; FC, fixation count; FFD, first fixation duration; FPRT, first-pass reading time; GD, gaze duration; IA, interest area (word-level AOI defined in eye-tracking software); ICC, intraclass correlation coefficient; LME, linear mixed-effects (model); LR, logistic regression; MLP, multilayer perceptron; NLP, natural language processing; OCR, optical character recognition; PSO, particle swarm optimization; RAN, rapid automatized naming; RF, random forest; RFE, recursive feature elimination; SA, saccade amplitude; SFD, single fixation duration; SI, self-intersection (gaze-trajectory event feature); SPRT, second-pass reading time; ST, speech therapy; STFT, short-time Fourier transform; SVM, support vector machine; TAU, treatment as usual; TFT, total fixation time; TRT, total reading time; TTFF, time to first fixation; VAS, vertical alteration score (gaze-feature metric in Vajs et al., 2023)/visual attention span (training construct; context-dependent usage in some literature); YARC, York Assessment of Reading for Comprehension.

**Table 2:** Eye-movement features used for dyslexia detection and reading behavior analysis.

Feature	Description	Studies reporting the feature (author-year)	Importance for dyslexia detection	Application in dyslexia research
Fixation metrics				
Fixation duration	Time (ms) the eyes remain stationary on a word/region (e.g., mean fixation, first-fixation duration, single-fixation duration)	Gran Ekstrand 2021; Rossier-Bisaillon 2025 (adults); Rossier-Bisaillon 2026 (moving-window children); Scaltritti 2019; Holmqvist Olander 2017; Le 2023; Park 2024; Raatikainen 2021; Appadurai 2021; Benfatto 2016; Pereira 2024 (SFD); Shalileh 2023; Vaitheeshwari 2024; Rello 2015; Vajs 2022; Svaricek 2025; Virlet 2024 (FFD); Peters 2021 (19–22, 25, 27, 31–33, 41–45, 48–51, 54)	Longer/variable fixations reflect higher decoding/lexical processing load and slower reading	Used as core discriminative feature (ML screening), typographic/condition effects, intervention responsiveness (pre-post changes)
Fixation count	Number of fixations (overall, per AOI/word, or frequency)	Scaltritti 2019; Holmqvist Olander 2017; Le 2023; Park 2024; Raatikainen 2021; Appadurai 2021; Pereira 2024 (FC); Shalileh 2023 (FC); Vaitheeshwari 2024; Rello 2015; Bhargavi 2020; Vajs 2022; Svaricek 2025 (baseline); Peters 2021 (20–22, 25, 27, 31–33, 44, 48–51, 54)	Higher fixation counts typically indicate inefficient decoding, rereading, or unstable attention allocation	Used for group comparisons (DD vs. controls), predictive models, and task/format optimization
Gaze duration	Sum of consecutive first-pass fixations on a word (often close to first-pass reading time/dwell time)	Rossier-Bisaillon 2025 (adults); Holmqvist Olander 2017 (dwell time); Pereira 2024 (FPRT/TFT); Virlet 2024 (GD); Vaitheeshwari 2024; Rello 2015 (reading time/visits); Shalileh 2023 (word-level totals); Bhargavi 2020 (fixation gaze duration) (25, 31–33, 42, 45, 49, 54)	Captures sustained processing time on a word; sensitive to lexical difficulty and decoding deficits	Used to quantify slowed lexical access and to show intervention effects (GD ↓ after effective training)
Saccadic movements				
Saccade length/amplitude	Distance moved between fixations (letters/pixels/degrees); shorter forward saccades often mean inefficient reading	Gran Ekstrand 2021; Rossier-Bisaillon 2025 (adults); Rossier-Bisaillon 2026 (moving-window children); Scaltritti 2019; Park 2024; Raatikainen 2021; Appadurai 2021; Benfatto 2016; Shalileh 2023 (first saccade amplitude); Vaitheeshwari 2024; Virlet 2024 (SA) (19, 20, 22, 27, 31, 33, 41–45)	Shorter saccades + unstable forward progression often accompany decoding difficulty	Used in ML models, perceptual-span experiments, and intervention evaluation (SA ↑ toward typical after effective therapy)
Regression rate	Proportion/count of backward eye movements or re-reading; sometimes operationalized via second-pass/“go-past” measures	Gran Ekstrand 2021; Rossier-Bisaillon 2025 (adults; regressive fixation probability); Park 2024 (regression ratio); Appadurai 2021 (regression summaries); Benfatto 2016 (regressive features); Pereira 2024 (SPRT/s-pass time); Shalileh 2023 (regression in/out, go-past/paths); İleri 2025 (EOG-derived regressions); Bhargavi 2020; Latifoglu 2021 (re-reading detection); Peters 2021 (regression count in RAN) (22, 25, 27, 31, 32, 42, 43, 46)	Regressions index comprehension/decoding breakdown and rereading strategies; frequently reported in dyslexic reading behavior	Used as diagnostic input features, to characterize reading behavior, and as EOG-based movement targets in AI pipelines
Gaze pattern analysis				
Scan path/gaze trajectory	Spatial sequence of fixations/saccades; often visualized as scanpaths/heatmaps or summarized as scanpath length/complexity	Holmqvist Olander 2017 (scanpaths); Le 2023 (scanpath + heatmap); Appadurai 2021 (scanpath length + scanpath images); Bhargavi 2020 (scanpath metrics); Vaitheeshwari 2024 (trajectory maps/saliency) (25, 27, 33, 49, 50)	Captures global reading strategy differences (looping, skipping, unstable line tracking)	Used for qualitative discrimination, feature engineering (scanpath length/density), and deep learning via trajectory/saliency images
Gaze transition metrics	AOI-to-AOI transitions (e.g., text↔picture switching) or transition matrices between regions/sentences	Holmqvist Olander 2017 (text↔picture transitions); Raatikainen 2021 (AOI transition matrices) (20, 49)	Detects attentional control/strategy (integration vs. distraction; search behavior)	Used to quantify multimodal integration (illustrations) and to build ML features from AOI transition matrices
Fixation fractal dimension	Complexity/irregularity of gaze trajectory (often computed from fixation traces)	Vajs 2022 (21)	Dyslexic reading tends to show higher spatial/temporal complexity and less efficient patterns	Used as an interpretable “gaze complexity” marker and ML input
Fixation intersection coefficient	Counts/normalizes self-intersections of fixation gaze lines (spatial looping complexity)	Vajs 2022 (intersection coefficient); Vajs 2023 (self-intersection events as triggers) (21, 28)	Captures rereading-like loops and spatial instability	Used as engineered interpretable features; can be computed without full fixation parsing (raw x-y)
Vertical alteration score (VAS)	Index of vertical instability (y-axis direction changes) around trigger events during reading	Vajs 2023 (28)	Sensitive to line-tracking instability and dysfluent scanning	Used as a single interpretable real-time screening feature (works even at lower sampling rates)
Physiological eye signals				
Blink rate/blink events	Blink count or rate during reading (often derived from EOG or eye-event streams)	İleri 2025 (blink rate); Bhargavi 2020 (blink events in pipeline) (25, 46)	Proxy for cognitive load/visual strain; sometimes differs in DD	Used as auxiliary indicator and to compare display/typeface effects; sometimes included in engineered feature sets
Eye-movement signal energy (EOG)	Signal-level energy/effort measure from EOG channels (e.g., integrated squared amplitude)	İleri 2025 (46)	Reflects intensity/effort of eye movement behavior during reading	Used to quantify cognitive load and as supportive biomarker alongside deep learning scalogram inputs

AOI, area of interest; DD, developmental dyslexia; EOG, electrooculography; ET, eye tracking; FC, fixation count; FFD, first fixation duration; FPRT, first-pass reading time; GD, gaze duration; RAN, rapid automatized naming; SA, saccade amplitude; SFD, single fixation duration; SPRT, second-pass reading time (re-reading time); TFT, total fixation time; VAS, vertical alteration score [feature in Vajs et al. (28)].

**Table 3:** Eye-tracking devices and experimental paradigms used in included studies.

Study	Eye-tracking/sensor device	Calibration method	Stimulus type	Experimental paradigm
Eye-tracking observational studies				
Gran Ekstrand et al. (43)	Tobii screen-based ET (120 Hz)	Not specified	Whole passage text	School-based passage reading with ET screening + neuropsych comparison
Rossier-Bisaillon et al. (42) (adult oral reading)	EyeLink 1,000 Plus (1,000 Hz) + synchronized audio	9-point calibration (reported)	Alouette-R standardized French text	Reading aloud; eye-voice span analyses
Scaltritti et al. (44)	EyeLink 1,000 Plus (1,000 Hz), chinrest	Re-calibration before each page (9-point/locations)	Screenshotted real webpages (text AOIs)	Silent webpage reading + navigation + comprehension questions
Holmqvist Olander et al. (49)	SMI RED250 (250 Hz)	5-point calibration + 4-point validation	Short expository texts ± illustration (ROIs: text vs. picture)	Text-only vs. text+picture; recall/MCQ; transition analysis
Le et al. (50)	Tobii 4C (90 Hz)	Calibrated twice/participant (details limited)	Vietnamese pseudowords/words + paragraph	Reading tasks + comprehension; heatmap/scanpath visualization system
Park et al. (22)	Galaxy Tab S5e + VisualCamp SeeSo (front-camera gaze estimation)	5-point calibration + verification step	Grade-appropriate tablet passages	Test–retest reliability of gaze-based reading metrics
Rossier-Bisaillon et al.(41) (children moving-window)	Gaze-contingent moving-window eye-tracking (device model not reported)	Not specified	Sentences with window restriction (baseline, 10/7/5/3-character windows)	Reading aloud under moving-window manipulation (perceptual span)
Machine learning/AI dyslexia reening and classification				
Raatikainen et al. (20)	EyeLink 1,000 (1,000 Hz)	Calibration + re-calibration if drift/error	“Question page” sentences (internet search task)	Trial-based reading; AOI transitions → ML classification
Appadurai and Bhargavi. (27)	Ober-2 ET (dataset-based)	Not specified	Passage reading + comprehension	Event-based features + ML/CNN representations
Nilsson Benfatto et al. (19)	Ober-2 goggle-based ET (100 Hz)	Manual gain setting per axis/eye	Printed short passage (10 sentences/8 lines)	Silent reading + comprehension; SVM-RFE classification
Nerušil et al. (26)	Ober-2 dataset (100 Hz)	No change needed - consistent	Same Benfatto reading text	CNN on holistic gaze time-series/spectrum
Pereira et al. (32)	SMI iView X HI-SPEED (1,250 Hz), chin/forehead rest	9–13-point calibration	Controlled text (word length/frequency), 3 slides	Silent reading; cognitive + eye metrics → predictive models (DD vs. ADHD-I vs. control)
Shalileh et al. (31)	EyeLink 1,000 Plus/Portable Duo (1,000 Hz)	Not specified	30 Russian sentences (+ some comprehension Qs)	Silent sentence reading; fixation + word-level IA metrics + demographics → AI
Vaitheeshwari et al. (33)	HTC Vive Pro + Tobii VR4 (120 Hz)	9-point VR calibration	VR multi-page reading text	VR reading + comprehension; eye + NLP (BERT) + CNN maps + fusion
Rello and Ballesteros (54)	Tobii 1750	Individual calibration	12 short texts × 12 fonts	Silent reading + comprehension control; SVM
İleri et al. (46)	EOG (BIOPAC MP-36), 100 Hz (horizontal/vertical)	Electrode placement (no camera calibration)	Turkish texts varied by font/typeface	Read-aloud; EOG features + CWT scalograms → CNN classification
Bhargavi and Jothi Prabha. (25)	Ober-2 dataset; external validation with Pupil Labs	Not specified	Reading text (inherited dataset)	Fixation/saccade/blink event features + ML; external validation reported
Latifoglu et al. (47)	EOG (BIOPAC MP36), 100 Hz	Electrode montage + filtering	5-line text	Reading; detect re-reading vs. line-skipping; STFT spectrograms → CNN
Vajs et al. (21)	SMI RED-m (120 Hz)	5-point calibration + validation (0.5°)	Serbian text under 13 color configurations	Silent reading; engineered spatiotemporal features → ML
Vajs et al. (28)	SMI RED-m (60 Hz; evaluated at 30 Hz)	Not detailed (same dataset family)	Same colored-text paradigm	Raw x-y features (SI/VAS) → interpretable ML + “real-time feedback” concept
Svaricek et al. (51)	SMI RED 250 (250 Hz)	9-point calibration (≤0.5° deviation)	3 texts (at-level, below-level, pseudo-text)	Read aloud 2 min per text; fixation visualisations → ResNet18
Intervention/training studies				
Virlet et al. (45)	EyeLink 1,000	Not specified	134-word French story + comprehension	Pre-post (9 months): Speech Therapy vs. Proprioceptive +Speech; eye outcomes (FFD/GD/SA)
Peters et al. (48)	Gazepoint GP3HD (150 Hz)	9-point calibration (before sessions/RAN)	Fruit Ninja training; RAN grid stimuli; YARC reading texts	RCT: AVG-R vs. AVG + (eye-control) vs. TAU; pre-post reading + RAN eye metrics

AOI, area of interest; AVG, action video game; AVG-R, action video game training with standard mouse control; AVG+, action video game training with eye-gaze cursor control; BERT, Bidirectional Encoder Representations from Transformers; CNN, convolutional neural network; CWT, continuous wavelet transform; DD, developmental dyslexia; EOG, electrooculography; ET, eye tracking; FFD, first fixation duration; GD, gaze duration; Hz, sampling frequency (samples/s); IA, interest area (word-level AOI defined in eye-tracking software); MCQ, multiple-choice questions; NLP, natural language processing; ROI, region of interest; RAN, rapid automatized naming; SA, saccade amplitude; SI, self-intersection (gaze-trajectory event feature); SMI, SensoMotoric Instruments; STFT, short-time Fourier transform; SVM, support vector machine; TAU, treatment as usual; VAS, vertical alteration score (gaze-feature metric in Vajs et al., 2023; feature used in gaze trajectory analysis).

**Table 4:** Machine learning models used for dyslexia detection.

Study	Input data/device	Reading or eye-movement task	Features used	Model type	Validation method (as reported by study)	Reported performance
Raatikainen et al. (20) (Finland)	Eye movement data (EyeLink 1,000, 1,000 Hz)	Internet search “question page” reading (10 trials)	Fixation duration/count; saccade amplitude/duration; AOI transition matrices; histogram features; RF-selected feature subset	RF feature selection → SVM (RBF) (class-weighted)	Repeated stratified 5-fold cross-validation with grid search for hyperparameter tuning	Best hybrid: Accuracy 89.7% ± 4.0, Recall 84.8% ± 14.0
Appadurai and Bhargavi (27) (India)	Gaze points from Ober-2 dataset	Passage reading + comprehension questions	Fixation/saccade features via I-DA & I-VA; PCA + RFE-CV; also, fixation/scanpath images for CNN	SVM variants; Hybrid kernel SVM-PSO; RF/AdaBoost/LGBM/XGBoost; CNN	Repeated random train/test splits (80/20) combined with 10-fold cross-validation	Best reported: Hybrid SVM-PSO ~ 96%, XGBoost ~95% (CNN ~ 87–88%)
Nilsson Benfatto et al. (19) (Sweden)	Ober-2 eye tracker (100 Hz)	Silent short passage reading + comprehension	168 low-level fixation/saccade parameters (progressive/regressive; horiz/vert; version/vergence)	Linear SVM + SVM-RFE feature selection	10-fold stratified CV, repeated 100×; feature selection inside folds	Accuracy 95.6% ± 4.5%, Sens 95.5%, Spec 95.7%
Nerušil et al. (26) (Slovakia)	Horizontal gaze x-coordinate (avg L/R) from Ober-2 dataset	Same reading text task (Benfatto dataset)	Holistic: trimmed reading segment; time-signal + interpolation; DFT magnitude spectrum	CNN2/CNN3/CNN4 (2–4 conv layers)	Repeated random subsampling with multiple evaluation runs (100 iterations; train/validation split)	Best: Accuracy 96.6% ± 2.9; TPR 97.8% ± 2.1; TNR 95.4% ± 4.1
Pereira et al. (32) (Portugal)	Eye tracking + cognitive scores (SMI iView X HI-SPEED, 1250 Hz)	Silent reading with word frequency/length manipulation	FC, SFD, FPRT, SPRT, TFT + cognitive tests; final predictors include Backwards Digit Span, Vocabulary, Coding, FC on long low-freq words	Multinomial logistic regression (SPSS)	No explicit validation strategy reported (overall classification accuracy provided)	81.4% correctly classified
Shalileh et al. (31) (Russia)	EyeLink fixation report + word-level IA metrics + demographics (1,000 Hz)	Silent reading of 30 Russian sentences (+ subset comprehension)	Fixation duration/X/Y; IA metrics (FFD, TRT, FC, skip, regressions, first saccade amp, etc.) + demographics	Multiple models tested; best typically MLP (also CNN/RF/GB/SVM/LR etc.)	Bayesian hyperparameter tuning with stratified cross-validation; final performance reported using stratified 10-fold CV	Best (fix+demo): F1 = 0.912 ± 0.002; ROC-AUC = 0.983 ± 0.000; Best (IA + demo): F1 = 0.934 ± 0.005; ROC-AUC = 0.986 ± 0.001
Vaitheeshwari et al. (33) (Taiwan)	VR eye tracking (HTC Vive Pro + Tobii VR4, 120 Hz) + text semantics + gaze maps	VR multi-page reading + comprehension	Fixation duration/count, saccade amplitude, gaze/trajectory metrics; saliency/trajectory maps; BERT text embeddings	SVM/RF/KNN/DT/NB/XGBoost/DNN + BERT + CNN + voting fusion	Validation approach not clearly specified; performance reported on pilot dataset	Fusion/voting model reported ~98% accuracy (pilot)
Rello and Ballesteros (54) (USA/Spain)	Tobii 1750 ET features + age	12 short texts × 12 fonts + comprehension control	Reading time (sum of visits), mean fixation duration, # fixations (+ age)	Polynomial SVM (LIBSVM)	10-fold CV keeping each subject’s readings in same fold	Accuracy 80.18% (subject-wise cross-validation applied)
İleri et al. (46) (Türkiye)	EOG (BIOPAC MP-36, 100 Hz) → CWT scalograms	Read-aloud Turkish texts varied by typeface/font size	Scalogram images (DL input); also reports blink rate/regressions/energy as indicators	Proposed DyslexiaNet CNN vs. AlexNet/ResNet50/MobileNetV2	5-fold CV (K = 5)	DyslexiaNet: ~99.968% accuracy (horizontal channel); vertical channel ~73.7% (performance should be interpreted with caution given dataset size and modality differences)
Bhargavi and Jothi Prabha (25) (India)	Ober-2 dataset + external Pupil Labs validation	Reading task (inherited dataset)	Fixation/saccade/blink event features; best subset includes avg. # fixations, avg. fixation gaze duration, avg. saccade duration, total # saccades	Hybrid kernel SVM-PSO + comparisons (SVM/RF/LR/KNN)	10-fold cross-validation with additional train/test split (80/20); external validation described	Hybrid SVM-PSO 95.6% accuracy; external validation ~96.6%
Latifoglu et al. (47) (Türkiye)	EOG (BIOPAC MP36, 100 Hz) → STFT spectrograms	Reading 5-line text; targets rereading vs. line-skipping events	Event segments converted to STFT spectrogram images	2D-CNN classifier	Validation strategy not clearly specified; performance evaluated on constructed dataset	Accuracy 99%, Sensitivity 100%, Specificity 98.18%, F-score 98.95% (event-type classification; not dyslexia classification)
Vajs et al. (21) (Serbia)	SMI RED-m ET (120 Hz)	Silent reading of 13 texts under 13 color configs	Conventional + proposed features (active reading time, fixation intersection coefficient/variability, fixation fractal dimension, saccade variability)	Logistic Regression, SVM, KNN, RF	Leave-one-subject-out cross-validation (30 folds)	Best: ACC = 0.94 (LR) using proposed features
Vajs et al. (28) (Serbia)	Raw x-y gaze coords (SMI RED-m 60 Hz; also 30 Hz)	Same colored-text reading paradigm	Single-feature models: SI-event feature, VAS-event feature, baseline reading time	Logistic Regression, SVM, KNN, RF	Leave-one-subject-out cross-validation with inner 5-fold cross-validation for hyperparameter tuning	Best: ACC 88.9% (VAS@60 Hz); best @30 Hz: ACC 87.8%
Svaricek et al. (51) (Czech Republic)	Fixation events (SMI RED 250, 250 Hz) → Fix-images	3 reading tasks (aloud 2 min each)	Fix-images built from fixation x/y + dispersion x/y + duration; baseline conventional metrics also tested	ResNet18 (fine-tuned) + majority-vote ensemble across tasks	Stratified 5-fold CV, repeated; cross-dataset test also reported	Ensemble Accuracy 86.65%; cross-dataset test 86.11%

Acc, accuracy; AUC, area under the receiver operating characteristic curve; CNN, convolutional neural network; CV, cross-validation; DD, developmental dyslexia; DFT, discrete Fourier transform; DL, deep learning; EOG, electrooculography; F1, F1-score; FC, fixation count; FFD, first fixation duration; FPRT, first-pass reading time; GD, gaze duration; KNN, k-nearest neighbors; LR, logistic regression; LSTM, long short-term memory (if reported); MLP, multilayer perceptron; NB, naïve Bayes; PSO, particle swarm optimization; RBF, radial basis function; RF, random forest; RFE, recursive feature elimination; ROC, receiver operating characteristic; SA, saccade amplitude; Sens, sensitivity; Spec, specificity; SFD, single fixation duration; SPRT, second-pass reading time; SVM, support vector machine; TFT, total fixation time; TNR, true negative rate (specificity); TPR, true positive rate (sensitivity); VAS, vertical alteration score.

**Table 5:** Standardized performance summary, target label quality, and risk of performance inflation (ML/AI studies).

Study	Sample size /dataset	What the model predicts	Model type	Validation/split	Standardized performance (as reported)	Target label quality	Risk of performance inflation
Raatikainen et al. (20)	*N* = 161 students; eye-movement dataset with reading-fluency cutoff labels	Poor readers/reading disorder vs. others	RF → SVM (RBF)	Stratified 5-fold CV (+ tuning); NEV	Acc 89.7%; Recall 84.8%	Screening proxy (reading-fluency cutoff label)	Moderate
Appadurai and Bhargavi (27)	*N* = 185 children; Benfatto eye-tracking dataset	Dyslexia risk (Benfatto dataset)	Hybrid SVM-PSO; XGBoost; CNN	Repeated random splits + CV; NEV	Best Acc ~ 96%	Screening proxy (high-risk vs. low-risk dataset label)	High
Nilsson Benfatto et al. (19)	*N* = 185 children; 97 high-risk and 88 low-risk readers	High-risk vs. low-risk	Linear SVM + SVM-RFE	10-fold CV repeated 100×; NEV	Acc 95.6% ± 4.5%; Sens/Spec ~95%	Screening proxy (risk label from reading tests/percentiles)	Moderate
Nerušil et al. (26)	*N* = 185 children; shared Benfatto dataset	High-risk vs. low-risk (same dataset)	CNN on holistic gaze signal/spectrum	100-fold eval (train/val described); NEV	Acc 96.6% ± 2.9%	Screening proxy (shared risk-labeled dataset)	Moderate
Pereira et al. (32)	*N* = 59 children; control, DD, and ADHD-I groups	Multi-class: DD vs. ADHD-I vs. Control	Multinomial logistic regression	Not clearly reported (no cross-validation or independent test set described)	81.4% correct	Clinical diagnosis (DD/ADHD-I group labels reported by authors)	High
Shalileh et al. (31)	*N* = 307 pupils; TD, DR, and DD groups	Multi-class TD vs. DR vs. DD	Multiple ML; best MLP	Bayesian tuning + stratified CV; final stratified 10-fold CV; NEV	F1 0.912–0.934; ROC-AUC 0.983–0.986	Mixed (DD + “reading disorder/low-risk” category in dataset)	Low
Vaitheeshwari et al. (33)	*N* = 14; 10 DD and 4 controls	DD vs. Control (pilot)	ML + BERT + CNN maps + voting fusion	Pilot reporting; validation unclear; NEV	Fusion Acc ~ 98%	Clinical diagnosis (case–control labels in pilot)	High
Rello and Ballesteros (54)	*N* = 97; 48 DD and 49 controls	Dyslexia vs. Control	Polynomial SVM	10-fold CV; subject-wise fold grouping; NEV	Acc 80.18%	Clinical diagnosis (case–control)	Moderate
İleri et al. (46)	*N* = 36; 23 DD and 13 controls	Dyslexia vs. Control (EOG)	CNN (DyslexiaNet) on CWT scalograms	5-fold CV; NEV	Acc ~ 99.97% (horizontal channel)	Clinical diagnosis (case–control)	High
Bhargavi and Jothi Prabha (25)	Benfatto dataset; reported 185–187 participants	Dyslexia risk (shared dataset)	Hybrid SVM-PSO	10-fold CV; external validation claimed; NEV	Acc 95.6%; external ~96.6%	Screening proxy (shared risk-labeled dataset)	High
Latifoglu et al. (47)	*N* = 20; 10 DD and 10 controls	Re-reading vs. line-skipping events (not dyslexia diagnosis)	2D-CNN on STFT spectrograms	Split method unclear; external validation not applicable because outcome was eye-movement event classification	Acc 99% (event classification)	Eye-movement event classifier (re-reading vs. line-skipping), not dyslexia diagnosis	Not comparable to dyslexia classification studies
Vajs et al. (21)	*N* = 30 children; 15 DD and 15 controls; 378 trials	Dyslexia vs. Control	LR/SVM/KNN/RF	Leave-one-subject-out CV: NEV	Best Acc 0.94	Clinical diagnosis (case–control label reported)	Moderate
Vajs et al. (28)	Same 30-child dataset; 15 DD and 15 controls	Dyslexia vs. Control	LR/SVM/KNN/RF (single-feature SI/VAS)	Leave-one-subject-out + inner CV: NEV	Best Acc 88.9% (60 Hz)	Clinical diagnosis (case–control label reported)	Moderate
Svaricek et al. (51)	*N* = 35 pupils; 13 DD and 22 controls	Dyslexia vs. Control	ResNet18 on Fix-images + ensemble	Stratified 5-fold CV repeated; cross-dataset test reported	Acc 86.65%; cross-dataset 86.11%	Clinical diagnosis (case–control)	Moderate

Acc, accuracy; ADHD-I, attention-deficit/hyperactivity disorder—predominantly inattentive presentation; AUC, area under the receiver operating characteristic curve; CNN, convolutional neural network; CV, cross-validation; DD, developmental dyslexia (dyslexia group); DR, reading disorder/low-risk or struggling reader group (as defined by the original dataset in that study); DFT, discrete Fourier transform; EOG, electrooculography; F1, F1-score; FC, fixation count; LR, logistic regression; MLP, multilayer perceptron; NLP, natural language processing; PSO, particle swarm optimization; RBF, radial basis function; RF, random forest; RFE, recursive feature elimination; SA, saccade amplitude; Sens, sensitivity; Spec, specificity; SVM, support vector machine; TD, typically developing (control group); TNR, true negative rate (specificity); TPR, true positive rate (sensitivity); VAS, vertical alteration score. Reported values were extracted from the included studies and were not recalculated. Unless otherwise stated, NEV indicates that model performance was evaluated within the original dataset only.

In addition, a structured synthesis was conducted to examine variability across studies in dataset labeling approaches (clinically validated vs. proxy labels), model validation strategies (internal, participant-wise, and external validation), and feature extraction pipelines, and to assess how these factors influence the interpretation and reliability of reported model performance. A comparative summary table (Table 6) was also developed to synthesize the main methodological approaches in terms of performance evidence, advantages, limitations, interpretability, and real-world feasibility. For modality-level synthesis, studies were additionally categorized as primarily eye-tracking/gaze-based studies, EOG-based eye-movement signal studies, or explicit multimodal/multi-source studies, defined as studies combining two or more independent input sources within the same analytical model.

**Table 6:** Comparative summary of methods for dyslexia screening and classification.

Methodological approach	Performance evidence	Main advantages	Main limitations	Interpretability and real-world feasibility
Conventional eye-tracking biomarker studies (41–44, 49)	Mainly group-difference evidence. Dyslexic readers generally showed longer fixations, more regressions, less efficient saccades, and slower reading patterns.	Directly measures reading behavior during text processing; useful for identifying gaze-based reading differences.	Does not establish screening accuracy by itself; affected by language, task, device, calibration, and feature definitions.	High interpretability; moderate feasibility because research-grade eye trackers may limit routine school use.
Eye-tracking-based ML/DL models (19–21, 25–27, 51, 54)	Reported accuracy ranged from about 80 to 96.6%. Examples include Rello and Ballesteros, 80.18%; Raatikainen et al., 89.7%; Nilsson Benfatto et al., 95.6%; Nerušil et al., 96.6%; Svaricek et al., 86.65% ensemble accuracy and 86.11% cross-dataset accuracy.	Can combine multiple gaze features and detect complex reading patterns; useful for automated screening/classification pipelines.	Risk of overfitting, proxy labels, small datasets, participant-level data leakage, and limited external validation.	Moderate interpretability for feature-based ML; lower for deep learning. Feasibility is moderate but requires stronger external validation before routine use.
EOG-based signal and deep-learning approaches (46, 47)	Latifoğlu et al. reported 99% 2D-CNN accuracy for classifying re-reading and skipping-line events. İleri et al. reported 99.96% accuracy for horizontal-channel EOG scalograms using DyslexiaNet.	Lower-cost physiological alternative to high-end eye trackers; captures regression, blink, reading-time, and EOG-energy indicators.	Requires electrode placement and signal-quality control; small samples; Latifoğlu et al. classified eye-movement events rather than dyslexia status.	Moderate interpretability for engineered EOG features; lower for CNN spectrogram/scalogram models. Feasibility is moderate in controlled settings but lower for schools.
Multimodal or multi-source models (31–33)	Pereira et al. reported 81.4% correct classification using eye-tracking plus cognitive predictors. Shalileh et al. reported F1 scores of 0.912–0.934 and ROC-AUC of 0.983–0.986 using eye-movement plus demographic/IQ data. Vaitheeshwari et al. reported about 98% accuracy in a small VR-fusion pilot study.	Combines complementary gaze, cognitive, demographic, linguistic, VR, BERT, CNN, or saliency-map features; supports personalized risk profiling.	Evidence remains exploratory; added modalities increase cost, complexity, missing-data risk, and validation requirements.	Interpretability varies: higher for feature-level fusion, lower for deep fusion. Current feasibility is moderate to low until larger externally validated datasets are available.
Tablet/front-camera and feasibility systems (22, 50)	Park et al. reported excellent reliability for fixation frequency, ICC = 0.83; fixation mean time, ICC = 0.82; reading speed by gaze, ICC = 0.76; and good reliability for regression ratio, ICC = 0.75, and saccadic length, ICC = 0.72. Le et al. tested 15 Vietnamese children using Tobii 4C at 90 Hz.	More scalable than laboratory systems; supports school or clinic workflows; outputs such as reading speed, fixation frequency, heatmaps, and scanpaths are easy to explain.	Reliability or feasibility evidence does not prove screening accuracy; sensitivity and specificity still need validation.	Moderate-to-high interpretability and high practical potential, especially for tablet-based systems, but clinical validation is still required.
Intervention response and monitoring studies (45, 48)	Evidence was limited to two studies. Peters et al. included 64 dyslexic children aged 8–13 years; Virlet et al. included 19 dyslexic participants plus controls.	Useful for monitoring within-person change and tailoring intervention support.	Small evidence base; heterogeneous interventions; not designed as standalone screening or classification models.	High interpretability; moderate feasibility if repeated testing protocols are standardized.

ML, machine learning; DL, deep learning; EOG, electrooculography; CNN, convolutional neural network; BERT, Bidirectional Encoder Representations from Transformers; VR, virtual reality; ICC, intraclass correlation coefficient; ROC-AUC, area under the receiver operating characteristic curve. Reported numerical values are taken from the included studies and were not recalculated.

## 3 Results

### 3.1 Study selection

Study identification and screening were conducted following the PRISMA 2020 framework (36). The database searches identified 1860 records in total – 1,215 from Scopus, 104 from PubMed/MEDLINE, 105 from CINAHL, and 436 from Web of Science. Ninety-nine non-English records were removed before screening, including 57 from Scopus, 11 from PubMed/MEDLINE, 7 from CINAHL, and 24 from Web of Science. After removal of duplicate records, database refinement, and preliminary exclusion of clearly ineligible records, 81 records remained for title and abstract screening. Of these, 31 records were excluded. A total of 50 full-text articles were sought for retrieval and assessed for eligibility, of which 23 studies met the inclusion criteria and were included in the qualitative synthesis. The complete selection procedure is illustrated in the PRISMA flow diagram (Figure 1).

### 3.2 Characteristics of included studies by evidence type

Key characteristics of the included studies are summarized in Table 1. The final evidence base consisted of 23 studies grouped according to study objective and evidence type: eye-movement biomarker/observational studies (*n* = 5), machine-learning/AI prediction-model studies (*n* = 14), intervention response studies (*n* = 2), and reliability or feasibility studies (*n* = 2). Two complementary studies by Rossier-Bisaillon et al. (41) were included: one examining eye-movement behavior during a standardized text-reading aloud task in dyslexic readers, and a second experimental study investigating perceptual span using a gaze-contingent eye-tracking paradigm in dyslexic children (42). Publication dates ranged from 2015 to 2026, reflecting the growing multidisciplinary interest in gaze-based approaches to dyslexia screening and assessment. This grouping was used because the included studies addressed different methodological questions: biomarker/observational studies primarily characterized dyslexia-related reading behavior, AI/ML studies evaluated prediction or classification performance, intervention response studies examined changes in eye-movement or reading outcomes following training or therapy, and reliability/feasibility studies evaluated measurement stability or practical implementation.

The studies were conducted across several regions, including Europe, Asia, and Australia, indicating the international scope of research in this field. Most investigations focused on children with diagnosed dyslexia or screened reading difficulties, although a smaller number of studies included adolescents or adults, such as those examining standardized oral reading with eye–voice alignment. Sample sizes differed considerably across studies, ranging from small experimental or pilot cohorts to larger datasets including more than 100 participants.

Reading paradigms also varied. Common experimental tasks included sentence or passage reading under silent or aloud conditions, standardized reading assessments, and more naturalistic tasks such as webpage reading. Some studies introduced controlled experimental manipulations, for example typographic variation, gaze-contingent moving-window paradigms, or color overlays. Across these designs, dyslexic readers were generally reported to display less efficient reading behavior, reflected in gaze-based measures and reading-performance indicators (e.g., reading rate and accuracy), although the magnitude and operationalization of these differences varied across tasks and participant groups.

### 3.3 Eye-movement biomarkers of dyslexia

Observational and experimental eye-tracking studies were synthesized as evidence for eye-movement biomarkers of dyslexia rather than as diagnostic prediction studies. A synthesis of eye-movement features reported across studies is presented in Table 2. Among both observational and prediction-model studies, fixation-based measures were the most commonly analyzed indicators of reading behavior. In many studies, dyslexic readers showed longer fixation durations, which are typically interpreted as reflecting increased cognitive processing demands during decoding and lexical access (43, 44). Measures related to re-reading behavior were also frequently examined. Several studies reported elevated regression activity among dyslexic readers, although the specific operationalization varied across investigations. Regression behavior was quantified using metrics such as regression probability, second-pass reading measures, or regression-path indices, all of which capture backward eye movements or rereading patterns during text processing (31).

In addition to fixation-related indicators, saccadic measures were widely reported. Compared with typical readers, dyslexic readers often demonstrated shorter or less efficient saccade amplitudes or lengths, consistent with slower progression through text and greater visual navigation demands (43, 45). Beyond these conventional features, several prediction-model studies incorporated higher-order representations of gaze behavior. Examples include area-of-interest (AOI) transition matrices (20), scanpath or trajectory-based representations, and measures of gaze complexity, such as fixation intersection metrics or fractal-dimension indices (21, 28). These features aim to capture broader patterns of spatial and temporal gaze organization during reading.

A smaller subset of studies relied on signal-based eye-movement measures derived from electrooculography (EOG) rather than camera-based eye tracking. In these studies, time–frequency representations and signal-derived indicators associated with rereading or visual effort were used as model inputs, thereby expanding the feature space beyond conventional fixation–saccade summaries (46, 47).

### 3.4 Eye-tracking devices and experimental paradigms

Information on devices and experimental paradigms is summarized in Table 3. Most studies employed screen-based infrared eye trackers in controlled laboratory or classroom settings, with reading material typically presented as sentences or passages on a monitor under silent or aloud reading conditions. Several investigations relied on high-frequency systems, such as EyeLink platforms, to capture fine-grained eye-movement events (20, 31, 41, 44, 45). Other studies explored more accessible configurations, including portable eye-tracking systems or tablet-based gaze-estimation approaches, to examine the feasibility of gaze-based screening tools (21, 22, 25, 26, 41, 45, 48).

Across the included studies, a variety of hardware platforms were used, including EyeLink devices, SMI trackers (49), Tobii screen-based systems (50), and goggle-based eye-tracking configurations such as the Ober-2 system in dataset-based work (19). Experimental paradigms also extended beyond conventional passage reading tasks. For example, some studies examined webpage reading or internet search behavior, whereas others incorporated controlled manipulations such as color overlays, typographic variations, or gaze-contingent moving-window paradigms designed to investigate perceptual span during reading. Two studies used electrooculography (EOG) rather than camera-based eye tracking to capture eye-movement signals during reading tasks. A smaller subset of research also explored immersive or virtual-reality reading environments with integrated eye tracking (33). Methodological procedures differed across studies, including calibration methods, sampling rates, stimulus presentation formats, and criteria for handling tracking loss or excluded trials. These methodological differences contribute to the overall heterogeneity observed across studies and may influence the comparability of eye-movement measures and derived features.

To clarify the use of the term multimodal, modality combinations were quantified across the 23 included studies. Most studies primarily used eye-tracking or gaze-derived reading features without explicit multi-source fusion. Explicit multimodal or multi-source modeling was identified in three of 23 studies. Shalileh et al. (31) combined eye-movement features with demographic and non-verbal intelligence variables; Pereira et al. (32) integrated eye-tracking data with cognitive and linguistic predictors; and Vaitheeshwari et al. (33) used a VR-based fusion approach combining eye-movement metrics with text-derived, saliency-map, and CNN-based representations. Two additional studies, İleri et al. and Latifoğlu et al., used EOG as an alternative eye-movement signal modality rather than as true multi-source fusion (46, 47). Therefore, the main evidence base remains dominated by eye-movement and gaze-based approaches, while multimodal evidence should be interpreted as emerging and exploratory.

### 3.5 Artificial intelligence/machine-learning (AI/ML) prediction models for dyslexia screening and classification

AI/ML studies were interpreted separately from observational biomarker studies because their primary objectives were to evaluate dyslexia-risk prediction, screening, or algorithmic classification performance rather than merely describe group differences in eye-movement behavior. Machine-learning and artificial-intelligence approaches used for dyslexia screening and algorithmic classification are summarized in Table 4, with a standardized overview of model performance and validation considerations presented in Table 5. Across the prediction-model studies, a range of algorithms were applied, including support vector machines, random forests, logistic regression, k-nearest neighbors, multilayer perceptrons, and deep-learning architectures, particularly convolutional neural networks (20, 21). The input data used in these models varied considerably, spanning conventional fixation- and saccade-based metrics as well as more complex representations such as AOI transition features, scanpath or trajectory encodings, gaze-complexity indices, and time–frequency representations derived from electrooculography (EOG) signals (28, 46).

Reported performance differed substantially across studies with classification accuracies typically ranging from approximately 80% to above 95%, and in some cases approaching 99%, particularly in studies using deep-learning models applied to signal-derived representations such as EOG-based inputs (46, 47). However, these high-performance estimates should be interpreted cautiously. Several studies used relatively small samples, internally derived datasets, or repeated observations from the same participants, which may increase the risk of overfitting if participant-level separation is not strictly maintained during model training and testing. In such circumstances, random train–test splits or conventional cross-validation may allow data from the same participant, reading trial, or recording session to influence both training and testing, leading to performance inflation. Direct comparison across studies is also limited by substantial heterogeneity in outcome definitions, feature engineering strategies, validation procedures, and reporting of model-development pipelines.

Important sources of variability included the nature of dyslexia labels and the choice of validation strategies. Across studies, datasets were classified as either (i) clinically validated datasets, in which dyslexia status was established through formal diagnostic procedures or (ii) proxy-labeled datasets, based on indirect criteria such as screening thresholds or dataset-derived groupings without explicit diagnostic confirmation. A substantial proportion of prediction-model studies relied on proxy labels, which may limit clinical validity and contribute to variability in reported model performance. Differences were also observed in model development and validation practices, including the use of participant-wise validation nested feature selection and hyperparameter tuning procedures, and the availability of independent or external test datasets (20, 21). Inadequate reporting or absence of these procedures makes it difficult to determine whether high reported accuracies reflect robust model performance or overfitting to a specific sample, device, language, or reading paradigm.

Model validation strategies can be broadly categorized into internal validation, participant-wise validation, and external validation using independent datasets. Internal validation methods, such as k-fold cross-validation or random train–test splits, were the most commonly used approaches across studies; however, these methods may lead to optimistic performance estimates, particularly in eye-tracking datasets where multiple observations from the same participant are included. In contrast, participant-wise validation, which ensures that all data from a given participant are confined to either training or testing sets, provides more realistic estimates of model performance by reducing the risk of data leakage. External validation represents the most rigorous approach for assessing generalizability but was employed in only a limited number of studies. Therefore, models reporting very high internal accuracy should be regarded as preliminary unless tested on independent participants, independent recording sessions, or external datasets collected using different devices, languages, or educational settings. Consequently, reported high accuracies-particularly those exceeding 95%-should be interpreted with caution when based solely on internal validation. The limited use of external validation, combined with inconsistent reporting of validation procedures and data partitioning, remains a key constraint in evaluating the robustness and real-world applicability of proposed models. Interpretation of model performance therefore requires careful consideration of study design, dataset characteristics, and validation rigor, as summarized in Table 5.

### 3.6 Intervention response studies

Intervention response studies were considered separately because their primary objective was to evaluate changes in reading or eye-movement outcomes following training or therapeutic intervention rather than to develop diagnostic prediction models. Two studies were classified in this category. Peters et al. (48) reported improvements in standardized reading outcomes following action video game training in children with dyslexia. In contrast, Virlet et al. (45) described gains in reading performance accompanied by changes in eye-movement measures after a proprioceptive-based intervention combined with speech therapy. These findings suggest that eye-movement measures may be sensitive to intervention-related change, but the small number of intervention studies and variability in intervention design limit conclusions regarding treatment effectiveness.

### 3.7 Reliability and feasibility studies

Reliability and feasibility studies were interpreted separately from biomarker, prediction-model, and intervention studies because their primary objective was to evaluate measurement stability, usability, or practical implementation of gaze-based screening approaches. Two studies were classified in this category. Park et al. (22) examined the test–retest reliability of a tablet-based dyslexia screening application using an eye-tracking system and reported good to excellent reliability for fixation-related metrics and gaze-based reading speed. Le et al. (50) evaluated an eye-tracking-based system for capturing visual reading strategies in children with dyslexia and described differences in scanpaths and fixation dispersion between dyslexic and typically developing readers. These studies support the feasibility of gaze-based screening approaches, but their findings should be interpreted as implementation and measurement evidence rather than as definitive diagnostic validation.

A structured comparison of the main methodological approaches is provided in Table 6, summarizing performance evidence, advantages, limitations, interpretability, and real-world feasibility across eye-tracking, EOG, machine-learning, multimodal, feasibility, and intervention-monitoring studies. Conventional eye-tracking studies provided interpretable evidence on reading behavior but did not independently establish screening accuracy. Eye-tracking-based machine-learning and deep-learning models showed stronger classification potential, whereas EOG-based approaches provided an alternative signal-based method for capturing reading-related eye movements. Multimodal models remained exploratory but may support personalized risk profiling, while tablet-based and low-cost gaze-estimation systems showed stronger practical feasibility for school or clinical workflows.

### 3.8 Risk-of-bias assessment

**Figure 2:**
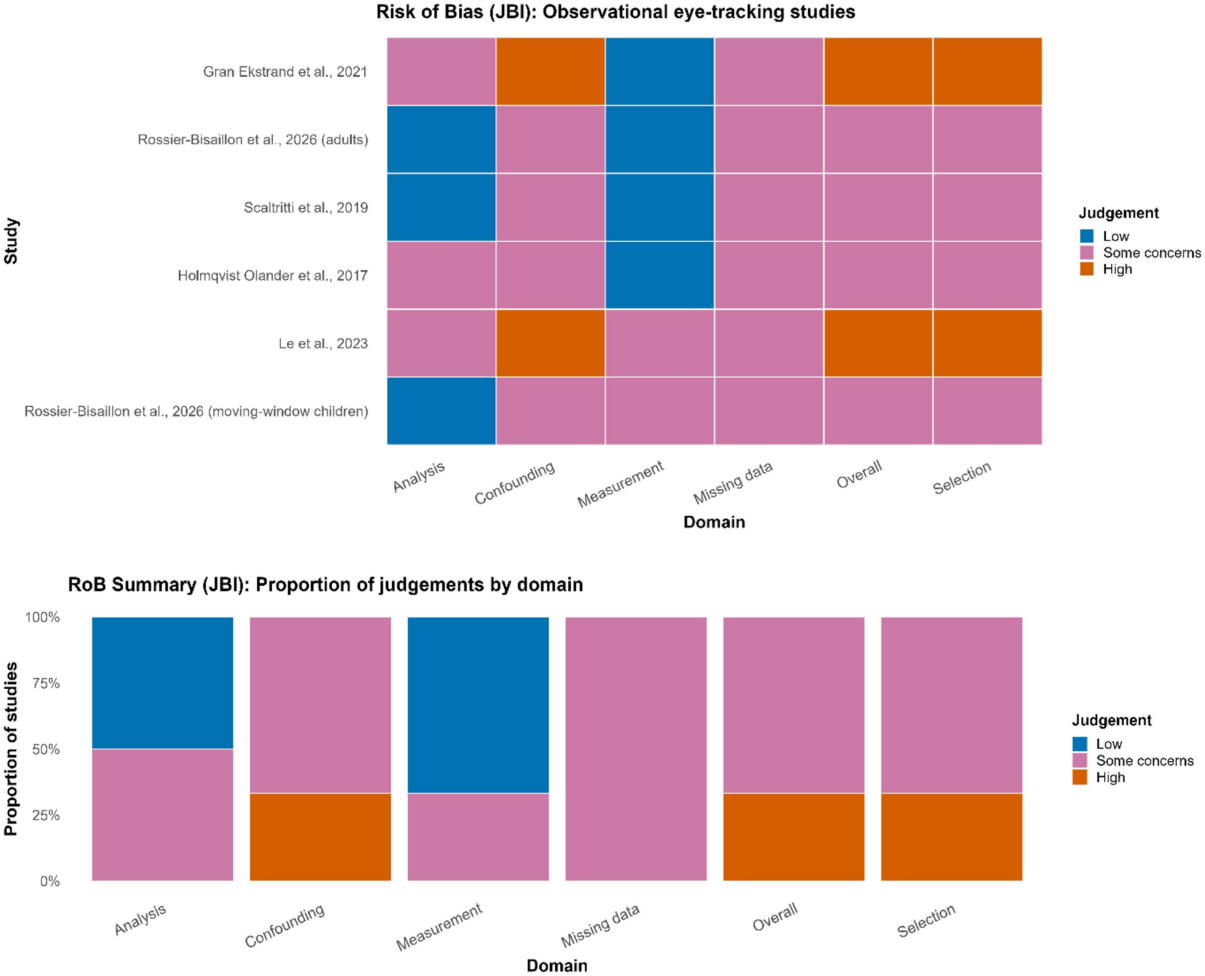
Risk-of-bias assessment for observational eye-tracking studies (JBI). The top panel presents a traffic-light plot summarizing domain-level judgments for each included observational eye-tracking study based on the Joanna Briggs Institute (JBI) critical appraisal framework. Domains include selection, confounding, measurement, missing data, analysis, and overall risk of bias. The bottom panel displays the distribution of studies across risk categories (low risk, some concerns, and high risk) for each domain.

Risk-of-bias findings were interpreted separately by evidence type because observational biomarker studies, prediction-model studies, intervention studies, and reliability/feasibility studies address different methodological questions and require different appraisal frameworks. Among the observational eye-tracking studies, six studies were appraised using the JBI checklist (Figure 2). Two studies were judged to be at high risk of bias (43, 50), primarily due to limitations related to participant selection and potential confounding factors. The remaining studies generally showed some concerns, with domain-level variability indicating relatively stronger ratings in measurement and analysis domains but more frequent concerns related to confounding control and selection processes. In contrast, measurement procedures and outcome definitions were typically reported more clearly.

**Figure 3:**
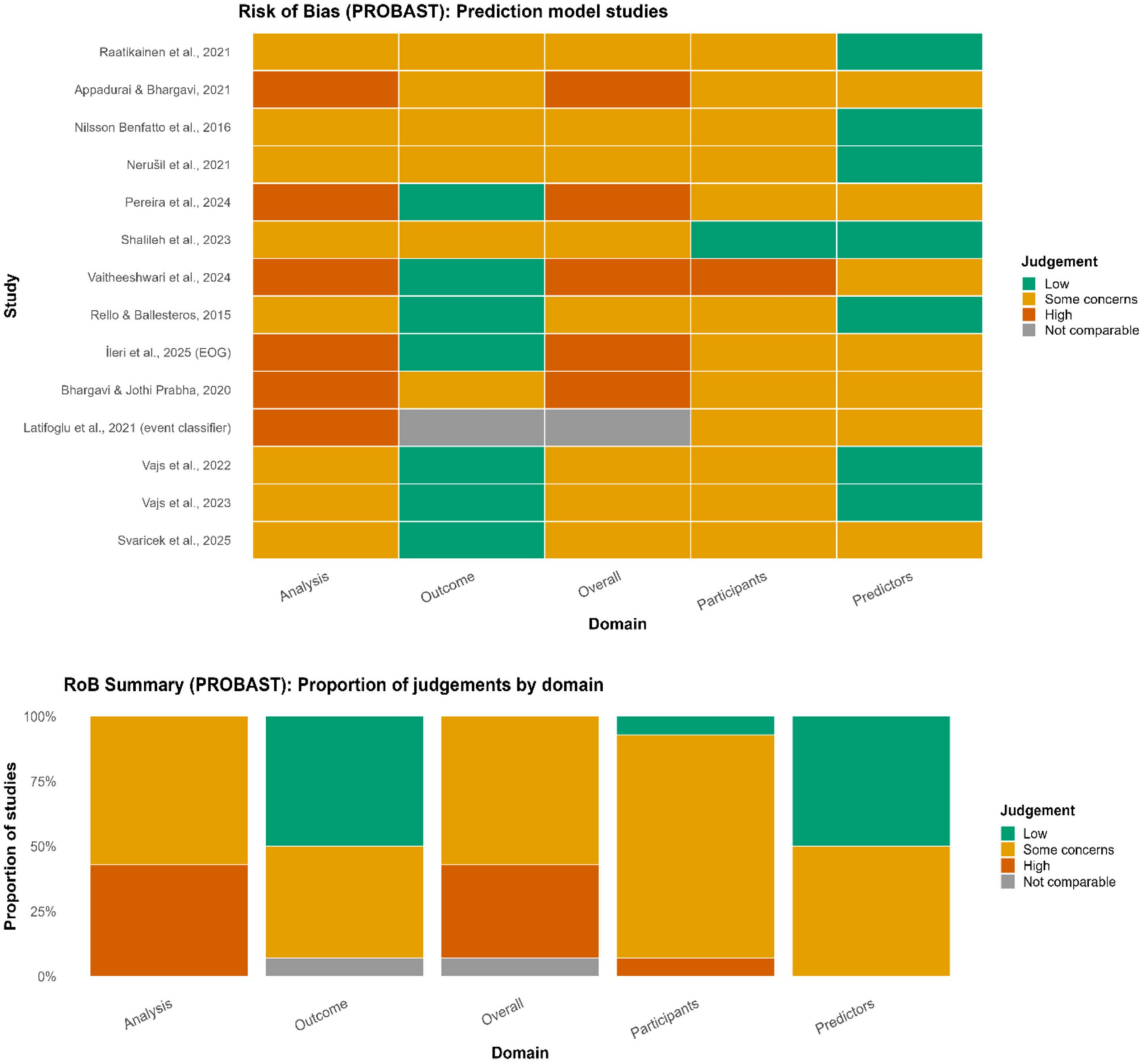
Risk-of-bias assessment for prediction model studies (PROBAST). The top panel shows a traffic-light plot summarizing domain-level PROBAST judgments for included machine-learning and AI prediction studies. Domains assessed include participants, predictors, outcome, analysis, and overall risk of bias. The bottom panel summarizes the proportion of studies classified as low risk, some concerns, high risk, or not comparable within each domain. The “not comparable” category was applied to studies in which the model outcome involved eye-movement event classification rather than dyslexia diagnosis.

The fourteen prediction-model studies were assessed using PROBAST (Figure 3). Most studies were categorized as some concerns, with a substantial subset rated high risk of bias, predominantly driven by limitations within the analysis domain. These concerns were particularly relevant to the interpretation of high reported accuracies because inadequate handling of participant-level data splitting, feature selection, hyperparameter tuning, and external validation can increase the risk of overfitting and performance inflation (19–21, 25–28, 51). Measurement of predictors was generally well described, but outcome definitions varied across studies, with several models relying on screening proxy labels rather than clinically confirmed diagnoses (19, 20, 25, 26). One study was not directly comparable, as it focused on classification of eye-movement event types rather than dyslexia status (47).

The two intervention or training studies, assessed using ROBINS-I, were judged overall as some concerns (45, 48). These ratings mainly reflected incomplete reporting of allocation procedures and potential deviations from intended interventions, although neither study was rated as high risk of bias overall. One study focused on test–retest reliability of gaze-based reading measures and was evaluated using COSMIN criteria (22). This study was judged as some concerns, primarily due to limited reporting of design elements relevant to reliability assessment (such as session standardization and exclusion criteria), although the statistical approach based on intraclass correlation coefficients (ICC) was considered appropriate.

## 4 Discussion

This systematic review synthesized evidence from 23 studies investigating dyslexia screening, risk identification, algorithmic classification, and reading characterization using eye-movement measures and computational approaches during reading-related tasks. Collectively, the findings indicate that eye-movement behavior provides quantifiable indicators of inefficient reading, although these measures reflect correlational rather than causal mechanisms. Across both observational and prediction-model studies, dyslexic readers consistently demonstrated longer fixation durations, increased regression behavior, and less efficient forward saccadic progression compared with typical readers, although the magnitude of these differences varied across experimental paradigms and populations (35, 52, 53). These patterns align with established interpretations of increased cognitive and perceptual processing demands during decoding and lexical access.

Because the included studies represented different evidence types, findings were interpreted according to study objective rather than as a single homogeneous evidence base. Observational and experimental eye-tracking studies provide evidence that dyslexic readers often show longer fixation durations, increased regressions, and less efficient saccadic progression during reading (41–44, 49). These findings are best interpreted as correlates or biomarkers of reading difficulty rather than diagnostic tests by themselves. In contrast, AI/ML prediction-model studies evaluate classification or screening performance, and their interpretation depends heavily on dataset labeling, feature extraction, validation strategy, and risk of overfitting (19–21, 25–28, 31–33, 46, 47, 51, 54). Intervention studies provide preliminary evidence that eye-movement measures may be sensitive to treatment-related changes, but the small number of such studies limits conclusions about intervention effectiveness (45, 48). Reliability and feasibility studies provide important information about measurement stability and practical implementation, but they do not provide the same level of evidence as externally validated diagnostic prediction models (22, 50).

Fixation-based and re-reading-related measures emerged as the most consistent indicators of reading difficulty. Fixation duration was the most frequently reported feature across both observational and prediction-model studies (31, 43, 44). Similarly, regression-related measures—operationalized through regression probability, second-pass reading indices, or regression-path metrics—were commonly elevated among dyslexic readers (31, 32, 41). These findings are consistent with theoretical accounts suggesting that disrupted decoding and lexical access increase reliance on reprocessing strategies during reading (55). Saccadic characteristics, particularly reduced saccade amplitude, further reflect inefficient forward progression through text and have been observed across both observational and intervention contexts (43, 45).

Beyond conventional eye-movement metrics, a notable trend is the increasing use of higher-dimensional representations of gaze behavior. Several studies incorporated scanpath-based features, area-of-interest (AOI) transition structures, and complexity-based metrics such as fixation intersection coefficients and fractal dimensions (20, 21, 28, 51). These approaches extend beyond single-feature descriptions and aim to capture the spatial and temporal organization of reading behavior, potentially providing a more comprehensive representation of underlying cognitive processes. Experimental paradigms such as gaze-contingent moving-window designs further contribute mechanistic insights, with evidence suggesting reduced parafoveal processing and increased reliance on foveal information in dyslexic readers (42).

A second major theme is the rapid expansion of machine-learning and deep-learning approaches for dyslexia screening, risk prediction, and algorithmic classification. The included prediction-model studies applied a wide range of algorithms, including support vector machines, random forests, logistic regression, multilayer perceptrons, and convolutional neural networks. Model inputs ranged from conventional fixation- and saccade-based features to more complex representations such as AOI transition matrices, fixation visualizations, and signal-domain features derived from electrooculography (EOG) (19, 20, 46, 51, 54).

Reported classification performance generally ranged from approximately 80% to above 95%, with some studies reporting values approaching 99%. These high values should be interpreted cautiously and should not be considered evidence of clinical readiness without rigorous validation. Several methodological factors may contribute to overfitting and performance inflation, including small sample sizes, repeated observations from the same participants, internal cross-validation without external testing, possible participant overlap between training and testing partitions, and limited reporting of nested feature selection or hyperparameter tuning. In eye-tracking datasets, this issue is particularly important because multiple trials, fixations, scanpath segments, or signal windows may be generated from the same participant. If these observations are randomly split rather than separated at the participant level, the model may learn participant-specific or session-specific characteristics rather than generalizable dyslexia-related reading patterns.

Differences in outcome definitions, feature engineering pipelines, and validation strategies also limit direct comparability across studies. Several investigations relied on proxy labels, such as high-risk versus low-risk groups, reading-fluency thresholds, or dataset-derived groupings rather than clinically confirmed dyslexia diagnoses (19, 25, 26). From an analytical perspective, this distinction is critical because proxy labels may not fully represent clinically defined dyslexia and may introduce label noise into machine-learning models. As a result, models trained on proxy-labeled datasets may demonstrate high internal accuracy but limited clinical generalizability. In contrast, studies using clinically validated labels and participant-wise or external validation provide stronger evidence for real-world applicability, but such studies remain comparatively limited. This imbalance represents a key challenge in translating gaze-based computational models into clinical or educational screening tools. In addition, many studies used internal cross-validation without independent external testing, increasing the risk of overfitting and optimistic performance estimates (56).

Validation strategy therefore plays a central role in determining the credibility of reported model performance. Internal validation approaches such as random train-test splits or k-fold cross-validation, may produce optimistic accuracy estimates when data are not separated at the participant level. Participant-wise validation provides a more robust estimate by ensuring that all observations from a participant are confined to either the training set or the testing set. External validation remains the strongest approach for assessing generalizability across different populations, languages, devices, and educational settings. The limited use of external validation, combined with small datasets and proxy labels, remains a major barrier to translating AI-based dyslexia screening systems into reliable clinical or educational tools. Model outputs in the included AI/ML studies should therefore be interpreted as screening or classification results rather than clinical diagnoses, unless dyslexia status was established through formal diagnostic assessment and the model was externally validated against clinically confirmed labels.

Substantial heterogeneity was observed in eye-tracking devices and experimental paradigms. While many studies used high-resolution, screen-based infrared eye trackers in controlled environments, others explored more ecologically valid settings such as webpage reading (44) or information-search tasks (20). Additional methodological diversity included gaze-contingent paradigms (42), EOG-based signal acquisition (46, 47), and immersive virtual-reality environments (33). These variations broaden potential applications but also introduce challenges for comparability, as differences in sampling rates, calibration procedures, and feature extraction pipelines can influence derived eye-movement metrics (49, 57). The comparative synthesis in Table 6 indicates that conventional eye-tracking measures offer stronger interpretability, whereas machine-learning, deep-learning, EOG-based, and multimodal approaches offer greater computational potential but require stronger validation, clearer reporting, and feasibility testing before routine implementation.

Evidence from intervention studies remains limited. Only two studies examined changes in reading behavior following targeted interventions. Peters et al. (48) reported improvements in standardized reading outcomes following action video game training, whereas Virlet et al. (45) observed improvements in reading performance accompanied by more typical eye-movement patterns following a proprioceptive-based intervention combined with speech therapy. Although these findings suggest that eye-movement measures may be sensitive to intervention-related changes, the small number of studies and variability in intervention design limit conclusions regarding effectiveness and underlying mechanisms.

From a precision educational healthcare perspective, the potential value of gaze-based and multimodal systems extends beyond binary screening or classification. These tools may support personalized risk profiling by identifying which reading-behavior features are most prominent in an individual learner, such as prolonged fixation duration, elevated regression activity, reduced saccadic efficiency, slow reading speed, or unstable scanpath organization. They may also support phenotype stratification by grouping learners according to dominant behavioral profiles, for example decoding-dominant difficulty, fluency-dominant difficulty, visual-attentional instability, oculomotor inefficiency, or mixed multimodal risk patterns. Such stratification could inform individualized intervention planning by helping educators and clinicians select support strategies that match the learner’s profile, rather than applying a uniform intervention approach to all children with reading difficulty. Repeated gaze-based assessment may also enable longitudinal monitoring of reading development and intervention response by tracking whether fixation duration, regression frequency, reading speed, or scanpath stability improves over time. However, these precision-medicine applications remain preliminary and require clinically anchored labels, interpretable models, longitudinal validation, and prospective evaluation in school and clinical pathways before routine implementation.

Risk-of-bias assessment further contextualizes these findings. Observational studies most frequently showed concerns related to participant selection and confounding, whereas measurement domains were generally stronger due to the objective nature of eye-tracking data. Among prediction-model studies, the analysis domain represented the primary source of bias, reflecting issues such as inadequate reporting of feature-selection procedures, lack of nested validation, and limited use of independent external datasets. These findings highlight a key challenge in AI-based screening research: high reported accuracy does not necessarily indicate robust or generalizable performance. It is also important to note that some studies, particularly those using EOG, focused on classification of eye-movement event types rather than dyslexia status (47). Although not directly comparable to dyslexia screening or classification models, such studies contribute to understanding of reading-related eye-movement dynamics.

Several limitations of this review should be acknowledged. First, substantial heterogeneity in study design and methodology precluded quantitative meta-analysis. This heterogeneity included variation in eye-tracking and EOG devices, sampling rates, calibration procedures, reading paradigms, stimulus presentation formats, and feature-extraction protocols, which may have affected the comparability of eye-movement measures and computational features across studies. Second, reliance on proxy diagnostic labels and internal validation in many prediction-model studies may limit clinical applicability. Third, cross-linguistic differences in orthography may influence eye-movement patterns and the diagnostic relevance of specific features (34). Finally, restriction to English-language peer-reviewed publications may have introduced language bias and may have excluded relevant evidence from non-English research contexts. In addition, because grey literature, dissertations, preprints, conference abstracts without full articles, and non-peer-reviewed reports were not searched, publication bias cannot be excluded, and studies with null, negative, or lower-performing results may be underrepresented. This restriction was applied for methodological feasibility because accurate eligibility assessment, data extraction, and interpretation of non-English studies would require appropriate language expertise and reliable translation resources. This is especially important because dyslexia and reading-related eye-movement behavior are influenced by orthographic and linguistic characteristics. Arabic orthographies, Chinese morphosyllabic writing systems, European shallow orthographies, and Indian multilingual populations may show different reading profiles, gaze patterns, and diagnostic feature relevance compared with English-language contexts. As a result, the generalizability of the findings to non-English and multilingual populations should be interpreted cautiously.

Future reviews should use multilingual search strategies and include non-English databases where feasible, and future model-development studies should prioritize cross-linguistic validation across diverse writing systems. From an applied perspective, gaze-based screening approaches may support early identification of reading difficulties in educational settings, particularly where access to specialist assessment is limited. However, real-world practice requires reliable and cost-effective eye-tracking or gaze-estimation hardware, robust calibration procedures, standardized reading-task protocols, and clear procedures for handling tracking loss or poor-quality recordings. Practical deployment also requires user-friendly systems that can be operated by non-specialist personnel and integrated into existing school or clinical referral pathways. While multimodal approaches may enhance individualized risk profiling, they may also increase system complexity, cost, and missing-data risk. Therefore, future implementation should balance predictive accuracy with usability, scalability, and feasibility in real-world educational and clinical contexts.

Future research should move toward technically robust and scalable model-development strategies. Transformer-based scanpath models may be useful for representing reading behavior as temporal sequences of fixations, saccades, regressions, fixation durations, and word-level interest-area transitions, rather than relying only on summary eye-movement features (58). Future studies should also explore multimodal fusion frameworks by combining gaze features with oral-reading speech signals, reading speed, decoding accuracy, cognitive test scores, and EOG/EEG-derived indicators. However, these approaches should be clearly distinguished from single-modality gaze-based models and EOG-based eye-movement signal models, because true multimodal fusion requires integration of two or more independent input sources within the same analytical model. Explainable AI methods should be incorporated so that model decisions can be interpreted using feature-importance approaches such as SHAP (59), or visual explanation methods such as Grad-CAM-like approaches for fixation maps, scanpath images, or time-frequency signal representations (60). Because screening tools may influence referral and educational support decisions, future models should also evaluate whether explanations are understandable and useful to clinicians, educators, and families (61, 62). Finally, privacy-preserving and deployment-oriented approaches, including federated learning across schools or clinics (63) and smartphone/tablet-based gaze screening with lightweight edge-compatible models (64), should be evaluated to improve scalability while protecting sensitive child-level educational and biometric data. These approaches align with digital phenotyping frameworks, in which repeated device-based behavioral signals can be used to monitor individual patterns over time and support data-driven risk profiling (65, 66). Future systems should be validated using clinically confirmed dyslexia labels, participant-wise and external validation, and cross-linguistic datasets before routine educational or clinical implementation.

## 5 Conclusion

This systematic review synthesizes current evidence on dyslexia screening, risk identification, and algorithmic classification using eye-movement measures and computational approaches during reading-related tasks. Across the available literature, dyslexic readers consistently demonstrate characteristic differences in gaze behavior, including longer fixation durations, increased regression activity, and less efficient forward saccadic progression. These patterns are generally interpreted as reflecting increased cognitive and perceptual demands during decoding and word recognition. Advances in eye-tracking technologies and computational methods have enabled the development of automated models that analyze gaze-derived features and higher-order representations of reading behavior. In addition, electrooculography-based approaches have been explored as alternative signal sources. Although reported screening and classification performance is often promising, interpretation remains constrained by substantial heterogeneity in study design, participant characteristics, outcome definitions, and validation strategies.

Future progress in this field will depend on greater standardization of experimental protocols and reporting practices, the availability of larger and more representative datasets, clearer specification of clinically confirmed outcome labels, and the implementation of rigorous validation strategies, particularly independent external testing. With continued methodological refinement and validation, gaze-based computational approaches have the potential to complement existing assessment methods and contribute to earlier, more objective, and scalable identification of individuals experiencing reading difficulties, while also supporting personalized risk profiling, intervention planning, phenotype stratification, and longitudinal monitoring within precision educational healthcare pathways.

## Data Availability

The original contributions presented in the study are included in the article/Supplementary material, further inquiries can be directed to the corresponding author.
