# Supplementary material for: AI-enabled eye-movement and emerging multimodal frameworks for precision dyslexia screening and reading pattern analysis
Source: Front Med (Lausanne). 2026 Jun 19;13:1847464. doi: 10.3389/fmed.2026.1847464 (PMC13328024; doi:10.3389/fmed.2026.1847464)
Supplement: Supplementary file 1 [file Table_1.docx]

**Supplementary File S1- Detailed Database Search Strategy**

**Title: Multimodal Vision Frameworks for Dyslexia Screening Using Eye-Movement and Reading Pattern Analysis: A Systematic Review**

**Supplementary Table S1. Database Search Strategies**

**1. PubMed Search Strategy**

The PubMed search combined Medical Subject Headings (MeSH) and free-text terms related to dyslexia, eye-movement analysis, and machine-learning approaches.

(

"dyslexia"[Mesh]

OR dyslexia[tiab]

OR "developmental dyslexia"[tiab]

OR "reading disorder"[tiab]

OR "reading difficulty"[tiab]

OR "reading difficulties"[tiab]

)

AND

(

"eye movements"[Mesh]

OR "eye tracking"[tiab]

OR "eye-tracking"[tiab]

OR "eye movement"[tiab]

OR "eye movements"[tiab]

OR gaze[tiab]

OR "gaze tracking"[tiab]

OR "gaze analysis"[tiab]

OR "gaze behaviour"[tiab]

OR "gaze behavior"[tiab]

OR oculomotor[tiab]

OR electrooculography[tiab]

OR EOG[tiab]

)

AND

(

"machine learning"[Mesh]

OR "artificial intelligence"[Mesh]

OR "machine learning"[tiab]

OR "artificial intelligence"[tiab]

OR "deep learning"[tiab]

OR "neural network"[tiab]

OR "predictive model"[tiab]

OR "prediction model"[tiab]

OR classification[tiab]

OR screening[tiab]

OR detection[tiab]

OR diagnosis[tiab]

)

**2. Scopus Search Strategy**

TITLE-ABS-KEY

(

dyslexia OR "developmental dyslexia" OR "reading disorder" OR "reading difficulties"

)

AND

TITLE-ABS-KEY

(

"eye tracking" OR "eye-tracking" OR "eye movement" OR "eye movements"

OR "gaze tracking" OR "gaze analysis" OR "gaze behaviour" OR "gaze behavior"

)

AND

TITLE-ABS-KEY

(

"machine learning" OR "artificial intelligence" OR "deep learning"

OR "neural network" OR "prediction model" OR "predictive model"

OR classification OR screening

)

AND

TITLE-ABS-KEY

(

reading OR text OR sentence OR word OR "reading task"

)

AND

PUBYEAR > 2014 AND PUBYEAR < 2027

AND

(LIMIT-TO (DOCTYPE, "ar"))

AND

(LIMIT-TO (LANGUAGE, "English"))

**3. Web of Science Core Collection Search Strategy**

- TS=(
  dyslexia OR "developmental dyslexia" OR "reading disorder"
  )
  AND
  TS=(
  "eye tracking" OR "eye movement" OR "gaze tracking"
  )
  AND
  TS=(
  "machine learning" OR "artificial intelligence" OR "deep learning"
  OR classification OR screening
  )
  AND
  TS=(
  reading OR text OR sentence
  )
  Timespan: 2015–2026
  Language: English
  Document type: Article

**4. CINAHL Search Strategy**

- (
- TI (dyslexia OR "developmental dyslexia" OR "reading disorder*" OR "reading difficult*")
- OR AB (dyslexia OR "developmental dyslexia" OR "reading disorder*" OR "reading difficult*")
- )
- AND
- (
- TI ("eye tracking" OR "eye-tracking" OR "eye movement*" OR "gaze tracking" OR "gaze analysis" OR "eye gaze" OR fixation* OR saccade* OR electrooculography OR electrooculogram OR EOG)
- OR AB ("eye tracking" OR "eye-tracking" OR "eye movement*" OR "gaze tracking" OR "gaze analysis" OR "eye gaze" OR fixation* OR saccade* OR electrooculography OR electrooculogram OR EOG)
- )
- AND
- (
- TX "machine learning"
- OR TX "artificial intelligence"
- OR TX "deep learning"
- OR TX "neural network*"
- OR TX classification
- OR TX classifier
- OR TX screening
- OR TX detection
- OR TX prediction
- OR TX "prediction model*"
- OR TX "predictive model*"
- OR TX reading
- OR TX "reading task*"
- OR TX "text reading"
- OR TX "word reading"
- OR TX "sentence reading"
- OR TX "reading speed"
- OR TX "reading fluency"
- OR TX "word recognition"
- )
